# Scalable and Sustainable Dry Microfabrication Enabled by High-Precision and Wafer-Scale Transfer Lithography of Commercial Photoresists

**DOI:** 10.1007/s40820-026-02215-7

**Published:** 2026-05-07

**Authors:** Qinhua Guo, Zhiqing Xu, Lizhou Yang, Jingyang Zhang, Yawen Gan, Jiajun Zhang, Jiahao Jiang, Yunda Wang

**Affiliations:** 1 Smart Manufacturing Thrust, The Hong Kong University of Science and Technology (Guangzhou), Guangzhou, 511400 People’s Republic of China; 2 Department of Mechanical and Aerospace Engineering, The Hong Kong University of Science and Technology, Kowloon, 999077 Hong Kong People’s Republic of China

**Keywords:** Photoresist transfer, Unconventional surface patterning, Reversible adhesion, Sustainable microfabrication, Phase-changing polymers

## Abstract

Conventional photolithography is inherently limited to flat, rigid, and stable substrates, which severely restricts its applicability to flexible, curved, and transient electronic devices. This work presents an innovative transfer method that exploits a phase-changing polymer with dynamically switchable adhesion to enable universal transfer of commercial photoresists onto a broad range of previously incompatible substrates, thereby overcoming the fundamental limitations of traditional photolithography. Remarkably, this method achieves reliable wafer-scale (~ 4-inch) transfer with a global registration error below 60 µm, unlocking high-fidelity patterning on challenging surfaces such as solvent-sensitive, curved, microtextured, or fragile substrates. Combined with dry etching, this study demonstrates a new route for high-resolution patterning of delicate functional materials, including quantum dots and organic semiconductors. Moreover, it supports a sustainable “dry lift-off” process for patterning functional layers, demonstrating a successful high-resolution microfabrication on paper-based substrate. The reusability of both the transfer carrier and photoresist markedly enhances process sustainability and scalability, representing a significant advance in microfabrication. This unprecedented capability is further demonstrated by fabricating a micro-sized UV photodetector array featuring wide-angle sensing capability on a curved glass bottle.

**Supplementary Information:**

The online version contains supplementary material available at 10.1007/s40820-026-02215-7.

## Highlights

- Universal, high-precision and wafer-scale (~ 4-inch) transfer lithography of multiple commercial photoresists for patterning various lithography-incompatible substrates.
- A novel route for etching-enabled high-resolution patterning of susceptible materials (e.g., quantum dots and organic semiconductors).
- A sustainable “dry lift-off” for functional material patterning and advanced device manufacturing (e.g., paper-based electronics and curved electronics).

**Supplementary Information:**

The online version contains supplementary material available at 10.1007/s40820-026-02215-7.

## Graphical abstract

**Figure:**
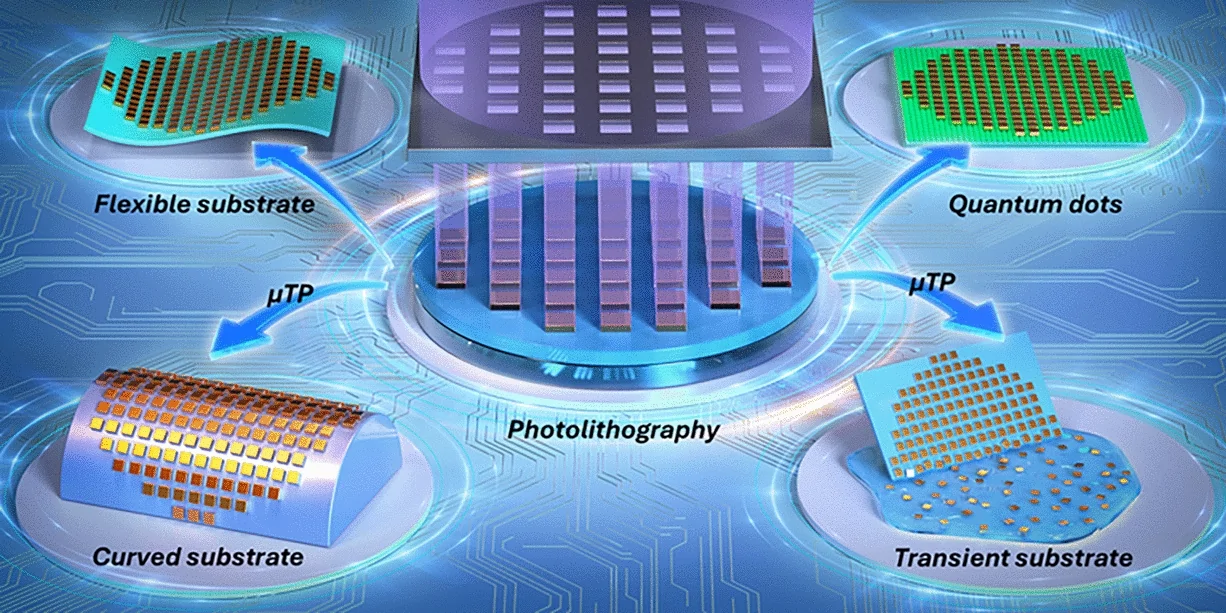

## Introduction

Photolithography remains the foundational technique for fabricating micro- and nanoscale features, enabling modern integrated circuits and micro-electro-mechanical system (MEMS) devices [1–4]. However, conventional photolithography is largely restricted to flat and rigid substrates, since any gaps or deformations in the resist layer or substrate can lead to light diffraction and compromise pattern fidelity [5]. Furthermore, the solvent-involved processes such as photoresist coating and development steps frequently cause swelling, dissolution, or chemical degradation of solvent-sensitive materials [6–8]. These limitations hinder high-precision patterning on unconventional substrates, such as curved or flexible surfaces, three-dimensional microtextured topographies, and delicate material layers including colloidal quantum dot films, where direct spin-coating and photoresist processing are often infeasible [7, 9–12]. Transfer printing of pre-fabricated functional inks has emerged as a transformative approach, enabling the integration of micro- and nanoscale materials onto a wide variety of unconventional substrates, including micro-/nanomaterial assembly [13, 14], flexible electronics [15, 16], curved electronics [17, 18], optoelectronics, and heterogeneous integration [19–22]. However, these ex situ fabrication methods also face challenges in terms of versatility and transfer reliability. Mechanical stress during the transfer process may damage delicate materials and devices, leading to serious performance degradation. Moreover, those methods are fundamentally incapable of performing in situ high-resolution microfabrication on materials that are inherently incompatible with conventional photolithographic workflows. Innovative approaches are therefore needed to extend lithographically defined high-resolution in situ microfabrication to these challenging contexts.

Photoresist transfer has emerged as a promising method for achieving high-resolution patterning on unconventional substrates. Several approaches have been developed, including detachment lithography [23], polydimethylsiloxane (PDMS)-based kinetic transfer [24, 25], and tape-assisted transfer [5, 26–28], each with distinct mechanisms and associated limitations. In detachment lithography, pattern definition is achieved through controlled mechanical fracturing of a continuous photoresist film [23]. In this method, a PDMS stamp coated with a uniform photoresist layer is brought into contact with a pre-patterned silicon master mold and then rapidly retracted to make the photoresist film broken along protruded features, leaving the desired patterned photoresist on the stamp. The patterned photoresist is then transferred to the receiver substrate via conformal contact followed by slow, controlled separation. Although detachment lithography can be applied to both planar and curved substrates, it requires pre-fabricated master molds with topography specifically tailored to the target features. Moreover, the abrupt mechanical retraction frequently induces irregular fracturing of the photoresist layer, which substantially limits process yield, reproducibility, and scalability. PDMS carrier-based approaches primarily exploit rate-dependent peeling dynamics to modulate adhesion of elastomer stamp, thereby enabling reliable photoresist transfer from low-surface-energy donor substrates onto unconventional receiver substrates [24]. Nevertheless, this velocity-controlled strategy carries inherent risks of deformation-induced fracture and insufficient adhesion contrast [29, 30]. Tape-assisted transfer employs thermal-release tapes to transfer custom-formulated photoresist, often incorporating surfactants to modulate photoresist adhesion to the donor substrate [5, 26, 27]. While method enables dry patterning on unconventional substrates, the reliance on customized photoresists may limit generality and scalability, making the approach less suitable for broader adoption. Furthermore, thermal-release tapes are inherently single use because of their irreversible adhesion transition, which severely restricts reusability in scalable production. Their limited conformability also restricts reliable contact on microtextured or non-planar surfaces. Crucially, transfer fidelity and wafer-scale registration have not been systematically demonstrated in these prior methods, leaving open challenges for precision-critical applications.

In this study, we report a sustainable and high-precision photoresist transfer method that relies on a phase-changing polymer with reversibly switchable adhesion. This polymer exhibits a huge storage modulus transition of nearly 2300-fold, providing strong adhesion and structural stability during the pickup phase while transitioning to a low-modulus state with high conformability during release phase, demonstrating an adhesion-switching ratio exceeding 110:1 for flat photoresist film. Consequently, the photoresist patterns are transferred with high fidelity and form excellent conformal contact with the receiver substrate. Both the transfer carrier and the photoresist can be reused multiple times, thereby establishing a scalable and sustainable platform for advanced microfabrication. Using this method, we demonstrate a high-precision and wafer-scale (~ 4-inch) photoresist transfer with a global registration error below 60 µm. It also enables the transfer of commonly used commercial photoresists, including AZ5214E and SU-8, onto a broad range of unconventional substrates, such as solvent-sensitive materials (e.g., cardstock paper and polyvinyl alcohol (PVA)), curved surfaces (e.g., cylindrical glass), microtextured surfaces with recessed cavities, and fragile materials (e.g., CsPbBr₃ quantum dots). Combined with dry etching, the method opens a new route for high-resolution patterning of solvent-sensitive materials such as quantum dots and organic semiconductors, with minimum feature sizes down to 5 µm. Furthermore, we establish a fully sustainable “dry lift-off” process for the direct patterning of metals and semiconductors on water-incompatible substrates (e.g., paper and PVA) and curved surfaces. As a practical demonstration, a functional 3 × 3 microscale UV photodetector array with wide-angle sensing capability was fabricated directly on the curved surface of a glass bottle.

Our approach represents a significant advancement in extending photolithography beyond planar surfaces, establishing a comprehensive and reusable framework for high-precision, solvent-free microfabrication with broad implications for flexible electronics, paper-based electronics, curved electronics, transient electronics, optoelectronics, MEMS, heterogeneous integration, and sustainable semiconductor manufacturing.

## Experimental Section

### Materials

Urethane diacrylate (UDA) was purchased from Sartomer Company, America. Stearyl acrylate (SA), trimethylolpropane triacrylate (TMP-TA), 2,2-dimethoxy-2-phenyl-acetophenone (DMPA), and benzophenone (BP) were obtained from Sigma-Aldrich, America. 3M 94 Primer (adhesion promoter) was acquired from 3M, America.

### Sharp Phase-Changing Rigid-to-Rubbery (SPRR) Polymer Preparation

#### FOTS-Treated Glass

A FOTS-treated glass is prepared by coating a monolayer of FOTS on a clean glass substrate through thermal evaporation in a chamber at 80 °C for 30 min.

#### SPRR Pre-polymer Preparation

The SPRR pre-polymer is customized utilizing a bistable electroactive polymer reported in previous study [31]. The pre-polymer solution is prepared by mixing SA (80 parts), UDA (20 parts), TMP-TA (1 part), DMPA (1 part), and BP (0.5 parts) in a glass bottle, followed by stirring at 80 °C. The SPRR polymers with different SA weigh fractions are fabricated by changing weight ratio of SA in SA-UDA copolymer. For example, the SPRR polymer (SA-60%) is fabricated by mixing SA (30 parts), UDA (20 parts), TMP-TA (1 part), DMPA (1 part), and BP (0.5 parts), where the weight ratio of SA to UDA is 60:40. SA-50% represents that the weight ratio of SA to UDA is 50:50. The SPRR polymer (SA-80%) is used as transfer carrier if there is no specific illustration.

#### 4-Inch SPRR Polymer/Polyethylene Terephthalate (PET) Carrier

First, a cleaned commercial PET film (thickness: 100 µm) is adhered to a cleaned 4-inch glass wafer. Then, an adhesion promoter layer (3M 94 Primer) is applied onto the PET film to enhance its surface adhesion. Following this, the liquid SPRR pre-polymer is injected into the gap between PET/glass substrate and a 4-inch FOTS-treated glass wafer, which are separated by spacers with a thickness of 350 µm. The SPRR pre-polymer is then solidified using 365 nm UV light at an intensity of 300 mW cm^−2^ for 3 min, followed by baking at 80 °C for 2 h to remove residual unreacted compounds. The SPRR polymer/PET carrier is subsequently obtained by removing two 4-inch glass wafers at 80 °C.

## Results and Discussion

### Working Principle of the SPRR Polymer-Based Photoresist Transfer Method

**Fig. 1:**
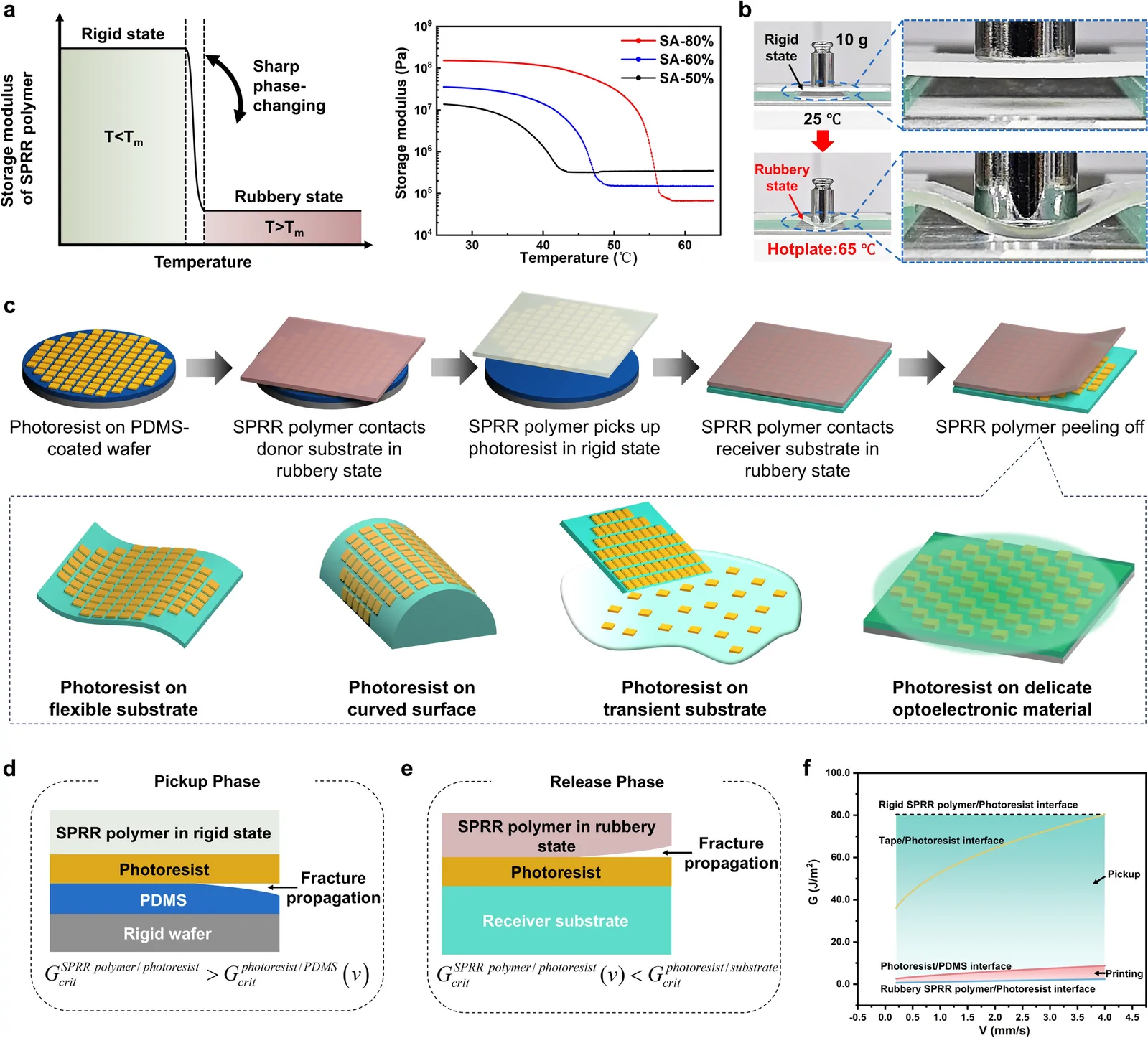
SPRR polymer-based photoresist transfer technology. **a** Dynamic mechanical analysis result of SPRR polymers with varied weight fractions of SA (50 parts, 60 parts and 80 parts) in the SA-UDA copolymers (100 parts). **b** Rigid-to-rubbery transition of SPRR polymer heated by a hotplate. **c** Schematic illustration of wafer-scale photoresist transfer process from PDMS-coated donor substrate to unconventional surface, utilizing the reversible rigid-to-rubbery phase transition of the SPRR polymer carrier. **d** Interface fracture competitions during pickup phase. **e** Interface fracture competitions during release phase. **f** Competition between different interfaces in transfer lithography

Figure 1 illustrates the working mechanism of the wafer-scale photoresist transfer method developed for patterning on non-traditional substrates. This method employs a sharp phase-changing rigid-to-rubbery polymer (SPRR polymer) to transfer commercial photoresists from low-surface-energy-treated donor substrates (e.g., PDMS-coated surfaces) to a variety of unconventional substrates [32], including those that are stretchable, flexible, curved, or otherwise susceptible. The SPRR polymer is primarily composed of stearyl acrylate (SA) and long-chain urethane diacrylate (UDA), where the SA crystalline aggregates serve as hard segments that markedly enhance the polymer’s stiffness and modulus in the rigid phase and be plasticizer in its molten state to soften SPRR polymer in rubbery state [33]. The long-chain UDA is used as the polymer framework to improve the toughness of the SPRR polymer in the rubbery state. As shown in Fig. 1a, this design enables the huge storage modulus change range of the SPRR polymer (80 parts SA in 100 parts SA-UDA copolymer) over a narrow phase change temperature range, exhibiting a large, reversible change from approximately about 66.7 kPa in rubbery state to 152.3 MPa in rigid state. To evaluate the reusability and potential performance attenuation of the SPRR polymer, dynamic mechanical analysis (DMA) was performed on SPRR polymer samples subjected to varying numbers of heating–cooling cycles. As shown in Fig. S1, the storage modulus remains on the same order of magnitude even after multiple thermal cycles, demonstrating the thermomechanical robustness of the SPRR polymer under repeated reuse conditions. The differential scanning calorimetry (DSC) measurements of SPRR polymer in Fig. S2 demonstrate a melting peak during heating and a recrystallization peak during cooling, where the melting temperature is determined as 43.6 °C. Figure 1b demonstrates the rigid-to-rubbery transition of SPRR polymer heated by a hotplate. The nearly 2300-fold changes in storage modulus fundamentally determine the adhesion-switching behavior of SPRR polymer. The detailed discussion about impact of SA and UDA on adhesion transition behaviors, and characterizations for loss modulus and tan δ of SPRR polymer are found in Section S1 of supplementary text and Fig. S3. This thermal responsiveness, accompanied with a shape memory polymer (SMP)-like behavior, enables controlled transitions between a soft, conformable state and a rigid, dimensionally stable state during the pickup and release steps. The low-surface-energy PDMS coating on the rigid donor substrate is applied via a scalable spin-coating process, while the transferable photoresist is prepared using standard photolithography.

Figure 1c shows the pickup/release protocols of transfer lithography, which involves fracture competition between two interfaces: the SPRR polymer/photoresist interface and the photoresist/substrate interface.

During the pickup phase, the SPRR polymer is heated above its melting temperature (*T* > *T*_m_) and brought into contact with the donor substrate in its rubbery state to achieve fully intimate contact with the photoresist. Upon cooling to room temperature, the SPRR polymer transitions into a rigid state while preserving the good contact, thereby forming a strong interfacial locking with the photoresist due to the higher interfacial modulus contrast [34]. As shown in Fig. 1d, in this state, the speed-independent critical energy release rate of the rigid SPRR polymer/photoresist interface $\left( {G_{{{\mathrm{crit}}}}^{{{\mathrm{Rigid}}\,{\mathrm{SPRR}}\,{\mathrm{polymer/photoresist}}}} } \right)$ exceeds the speed-dependent value of photoresist/PDMS interface $\left( {G_{{{\mathrm{crit}}}}^{{\mathrm{photoresist/PDMS}}} \left( v \right)} \right)$ when the SPRR polymer is retracted from the donor substrate at an appropriately low speed. Thus, the fracture occurs preferentially at the photoresist/PDMS interface.

During the release phase, the SPRR polymer carrying the photoresist is brought into contact with the receiver substrate and reheated above *T*_m_. As shown in Fig. 1e, when the SPRR polymer is retracted from the receiver substrate at an appropriately low speed, the speed-dependent critical energy release rate of rubbery SPRR polymer/photoresist interface $\left( {G_{{{\mathrm{crit}}}}^{{{\mathrm{Rubbery}}\,{\mathrm{SPRR}}\,{\mathrm{polymer/photoresist}}}} \left( v \right)} \right)$ is lower than that of photoresist/substrate interface $\left( {G_{{{\mathrm{crit}}}}^{{\mathrm{photoresist/substrate}}} } \right)$. Thus, the photoresist can be released onto the receiver substrate. This mechanism demonstrates the adhesion switching ratio of pickup to release on non-structured, flat-surface photoresist film higher than 110:1. Overall, the interface fracture competition mechanism in transfer lithography is schematically illustrated in Fig. 1f, where the rigid SPRR polymer/photoresist interface is expected to exhibit speed-independent energy release rate. Additional detailed discussion about transfer mechanisms, adhesion characterization, and effects of SPRR polymer’s material composition on adhesion transition behaviors is provided in Sections S1-S3 of supplementary text and Figs. S4–S10.

### High-Precision, Wafer-Scale Photoresist Transfer

**Fig. 2:**
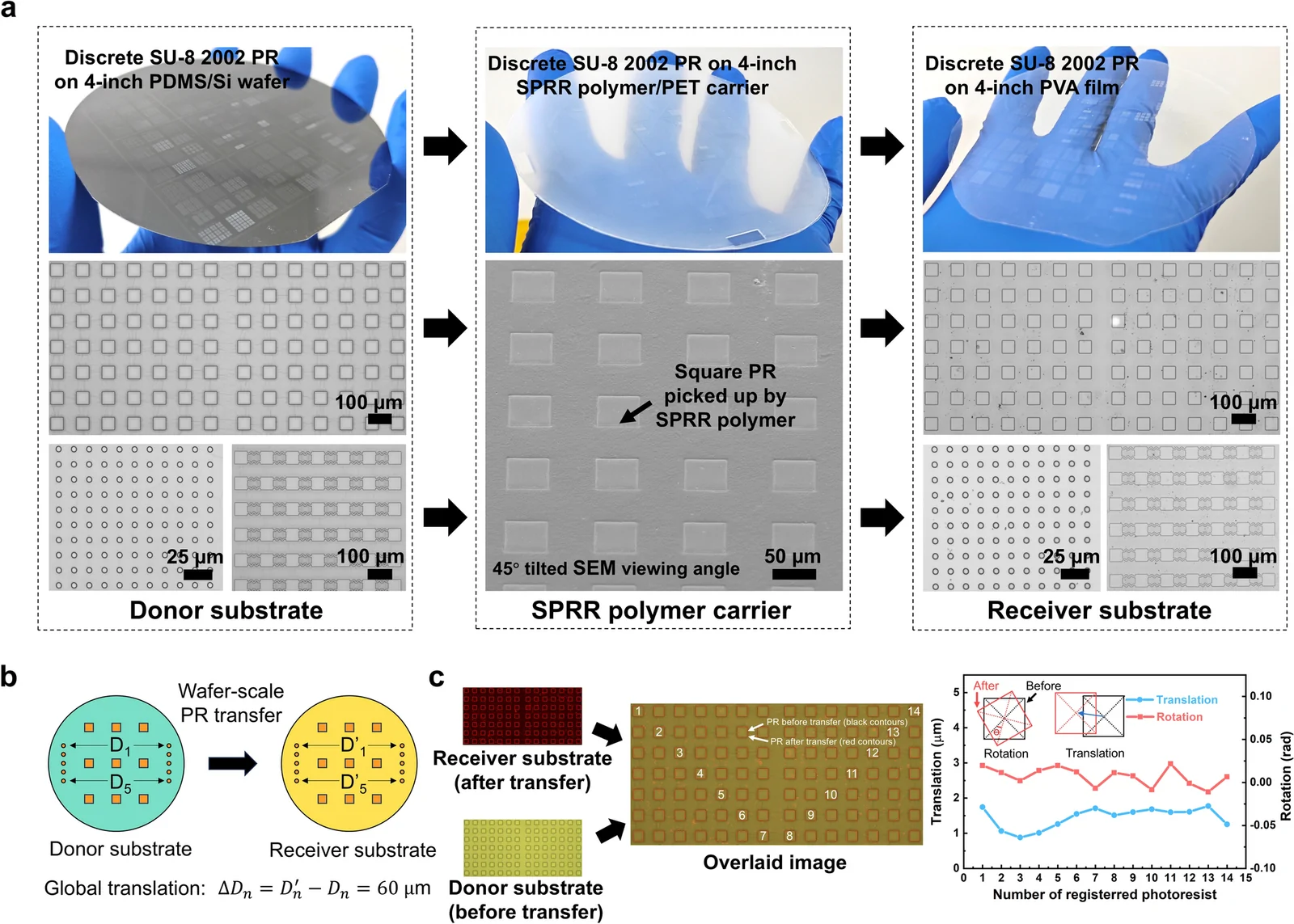
High-precision, wafer-scale photoresist transfer enabled by reversible adhesion transition. **a** Photographs and optical microscopy images showing key stages of the wafer-scale transfer process. Discrete multiscale SU-8 2002 photoresist structures were transferred from a 4-inch PDMS/silicon donor wafer to a 4-inch SPRR polymer/PET carrier (45° tilted SEM image) and subsequently to a 4-inch PVA receiver substrate. **b** Schematic illustration of global transfer error analysis before and after transfer. **c** Overlaid processed image of photoresist structures before and after transfer, along with analysis of local registration errors in translation and rotation

To evaluate the effectiveness and scalability of our method for patterning unconventional surface at the wafer scale, we conducted an experiment involving the transfer of photoresist from a 4-inch PDMS-coated wafer onto a 4-inch solvent-susceptible substrate which is PVA. As shown in Fig. 2a, multiscale patterns (5–50 µm features) of 1-µm-thick SU-8 2002 photoresist were photolithographically defined on a 4-inch PDMS-coated silicon wafer (PDMS thickness: 15 µm). A 4-inch SPRR polymer/PET carrier (350-µm-thick SPRR polymer coating on 100-µm-thick PET) was laminated onto the donor wafer using a commercial hot laminator. During the lamination process, the flexible SPRR polymer made intimate contact with the 4-inch donor wafer, while the smooth PET film minimized a shear force from the roller. After cooling to room temperature, the photoresist was picked up by separating the carrier at an average fracture propagation speed less than 2 mm s^−1^. For photoresist release, the SPRR polymer/PET carrier with photoresist was laminated onto a 4-inch, 100-µm-thick PVA film and peeled away on an 80 °C hot plate at the average fracture propagation speed less than 2 mm s^−1^, completing the transfer. Optical microscopy images confirmed successful transfer of multiscale patterns onto the PVA substrate. Global transfer error was analyzed using reference marks at opposite edges of the wafer as illustrated in Fig. 2b. The translation error was 60 µm across 84.94 mm, corresponding to a shift of ~ 0.07%.

Figure 2c shows an overlaid processed image of the discrete photoresist structures before and after the wafer-scale transfer. The alignment was achieved using a linear transform with assistance of Fiji software [35, 36]. The local registration error, measured as deviations in relative position of photoresist structures, was within 1.8 ± 0.9 µm for translation and below 0.03 ± 0.03 radians for rotation across a 1380 µm × 650 µm area. As another demonstration, Fig. S11 shows the wafer-scale transfer of AZ5214E photoresist onto a silicon wafer. The registration error for multiple points across the 4-inch wafer shows the maximum translation error lower than 1.9 µm. To our knowledge, these registration results represent the highest accuracy reported to date for photoresist transfer. This is attributed to the high modulus of the SPRR carrier in its rigid state, which effectively locks the photoresist structures before release, and its soft state, which enables damage-free release. To evaluate the effect of thermal history of SPRR polymer on the transfer registration accuracy, several SPRR polymer samples that had undergone 10 and 50 heating–cooling cycles were used to transfer SU-8 2002 photoresist from PDMS/silicon substrate to silicon substate. As shown in Fig. S12, pattern registration accuracy remains good and comparable to that achieved with pristine SPRR polymer, indicating tiny influence from prior thermal history.

Additional details regarding material preparation, the wafer-scale photoresist transfer process, registration method, and error analysis are provided in Experimental Section and supplementary text.

### Transfer of Commercial Photoresists onto Diverse Unconventional Substrates

To demonstrate the general applicability of our method, we conduct a series of experiments transferring various commercial photoresists onto different receiver substrates. The transfer protocol followed the same framework, comprising pickup and release steps. The donor substrates were prepared by patterning commercial photoresists via photolithography on PDMS-coated wafers. In these experiments, the SPRR polymer/glass carrier with a 1-mm-thick SPRR layer was used, and the heating step was performed on an 80 °C hot plate. The average fracture propagation speeds during pickup and release were estimated to be below 2 mm s^−1^.

**Fig. 3:**
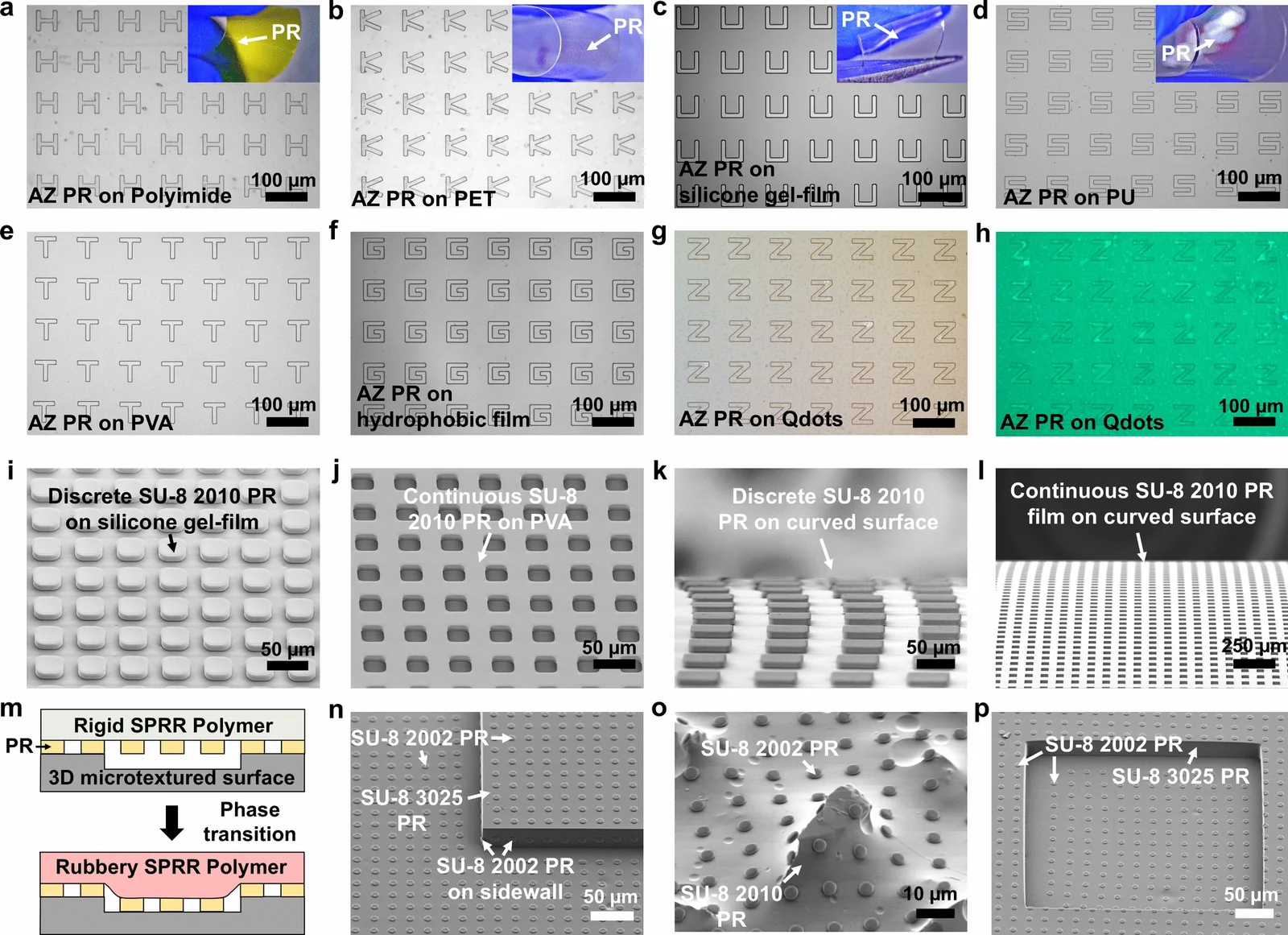
Photoresist transfer onto diverse unconventional surfaces. **a-d** AZ5214E photoresist structures transferred onto flexible substrates, including polyimide, PET, silicone gel film, and PU. **e—f** AZ5214E photoresist transferred onto substrates incompatible with conventional solvent-based lithographic processes, including water-soluble PVA and low-surface-energy fluoropolymer film. **g—h** Brightfield and fluorescence images of photoresist transferred onto CsPbBr_3_-coated glass substrates. **i** Discrete SU-8 2010 photoresist structures transferred onto a silicone gel film. **j** Continuous SU-8 2010 photoresist film transferred onto a PVA film. **k—l** Discrete structures and continuous film of SU-8 2010 photoresists transferred onto curved convex surfaces. **m** Schematic illustration of intimate contact between rubbery SPRR polymer carrier and pre-structured 3D topographies. Discrete 1-µm-thick SU-8 2002 photoresist structures transferred onto **n** PDMS substrates with 27-µm-high SU-8 3025 steps, **o** SU-8 2010 photoresist film with sandpaper-replicated protrusions and **p** PDMS substrates with 25-µm-deep recessed cavity formed using SU-8 3025 photoresist

Figure 3a–h shows 3-µm-thick discrete AZ5214E photoresist (AZ PR) structures with 10 µm feature sizes transferred onto a variety of unconventional substrates. Figure 3a–d illustrates successful photoresist transfers onto several flexible films, including polyimide (PI), polyethylene terephthalate (PET), silicone gel film, and polyurethane (PU). Additionally, the method is compatible with substrates that are incompatible with conventional solution-based lithographic processes. Figure 3e, f shows photoresist transfers onto a water-soluble PVA film and a fluoropolymer film with a hydrophobic surface, respectively. Figure 3g, h shows brightfield and fluorescence images, respectively, of AZ5214E photoresist transferred onto a CsPbBr_3_ quantum dots/glass substrate.

The method also supports transfer of thick photoresists. Figure 3i shows the SEM image of discrete 10-µm-thick SU-8 2010 photoresist structures transferred onto a free-standing silicone gel film. In addition to discrete features, the method enables transfer of continuous photoresist films. Figure 3j shows the SEM image of 10-µm-thick continuous film of patterned SU-8 2010 photoresist transferred onto a free-standing PVA substrate. Extending photolithography to curved surfaces has been a long-standing challenge due to the incompatibility of rigid masks and spin coatings with non-flat geometries [37, 38]. Using the SPRR polymer-based transfer method, we successfully demonstrated high-resolution photoresist patterning on curved convex surfaces. Figure 3k, l shows discrete structures and a continuous film of SU-8 2010 photoresist, respectively, transferred onto curved substrates using a 1-mm-thick free-standing SPRR polymer carrier. Further details on material preparation and photoresist transfer processes are provided in Experimental Section.

The SPRR polymer with 80% SA content exhibits the largest storage modulus transition and therefore the strongest reversible adhesion switching, enabling reliable pickup and release across a broad range of commercial photoresists, including SU-8 2002, SU-8 2010, and AZ5214E. In contrast, polymers with lower SA contents (50% and 60%) display reduced modulus contrast and consequently weaker adhesion-switching strength. As a result, their applicability depends more sensitively on the intrinsic adhesion strength of the photoresist to the donor substrate. For instance, SU-8 2010, which exhibits relatively strong adhesion to the donor surface, cannot be effectively picked up using the 50% SA formulation and shows limited yield under high-speed pickup with the 60% SA formulation. By comparison, AZ5214E and SU-8 2002, which have comparatively lower adhesion to donor substrates, can be successfully handled by all three formulations.

### Patterning on Pre-structured 3D Topographies

As illustrated in Fig. 3m, a key advantage of the SPRR polymer carrier lies in its ability to transfer photoresist onto pre-structured 3D topographies. To demonstrate this capability, we used the SPRR polymer/glass carrier to transfer photoresists onto a variety of substrates with distinct 3D features. SU-8 2002 photoresist patterns were first defined on PDMS/silicon donor wafers and picked up by the SPRR carrier using the standard pickup protocol. During this process, the SPRR polymer and the donor substrate were pre-heated on an 80 °C hot plate to ensure intimate contact, while pickup was conducted at room temperature with an average fracture propagation speed below 2 mm s^−1^. Photoresist release was then carried out using a six-axis stage (Fig. S13), with the SPRR polymer/glass carrier heated by a heat gun and retracted at a controlled speed of 5 µm s^−1^. Figure 3n shows 1-µm-thick, 10-µm-diameter SU-8 2002 structures successfully transferred onto a PDMS substrate containing 27-µm-high SU-8 3025 step structure. The transferred SU-8 2002 photoresist conformally covered the underlying 3D profile, facilitated by the intimate contact between receiver substrate and rubbery SPRR polymer during the release phase. Figure 3o shows a 1 cm × 1 cm array of 5-µm-diameter SU-8 2002 photoresist transferred onto a SU-8 2010 photoresist/glass substrate with randomly protruded morphology. The rough morphology was formed through double casting procedures, where the substrate was casted from a sandpaper-molded PDMS template, thereby replicating the microstructures present on the sandpaper (P800, grain size: 21.8 µm). Additional optical images in Fig. S14 show that the pattern integrity in this demonstration is largely preserved in the flat regions between local protrusions, while localized distortions may occur near steep or abrupt features. These distortions are related to the local mismatch between the pattern geometry and surface curvature and are expected in such highly irregular 3D contexts. The transfer pattern with local distortion also demonstrates statistical regularity, leaving potential applications in some contexts, such as 3D hierarchical micro-structure manufacturing. Figure 3p demonstrates successful transfer of SU-8 2002 photoresist onto a PDMS substrate with a 25-µm-deep recessed cavity formed using SU-8 3025 photoresist. These results demonstrate the unique ability of our method to pattern over complex 3D surfaces, which remains infeasible for conventional photolithography and previously reported photoresist transfer techniques. Additional experimental details are provided in Experimental Section.

### High-Resolution Patterning of Susceptible Materials

**Fig. 4:**
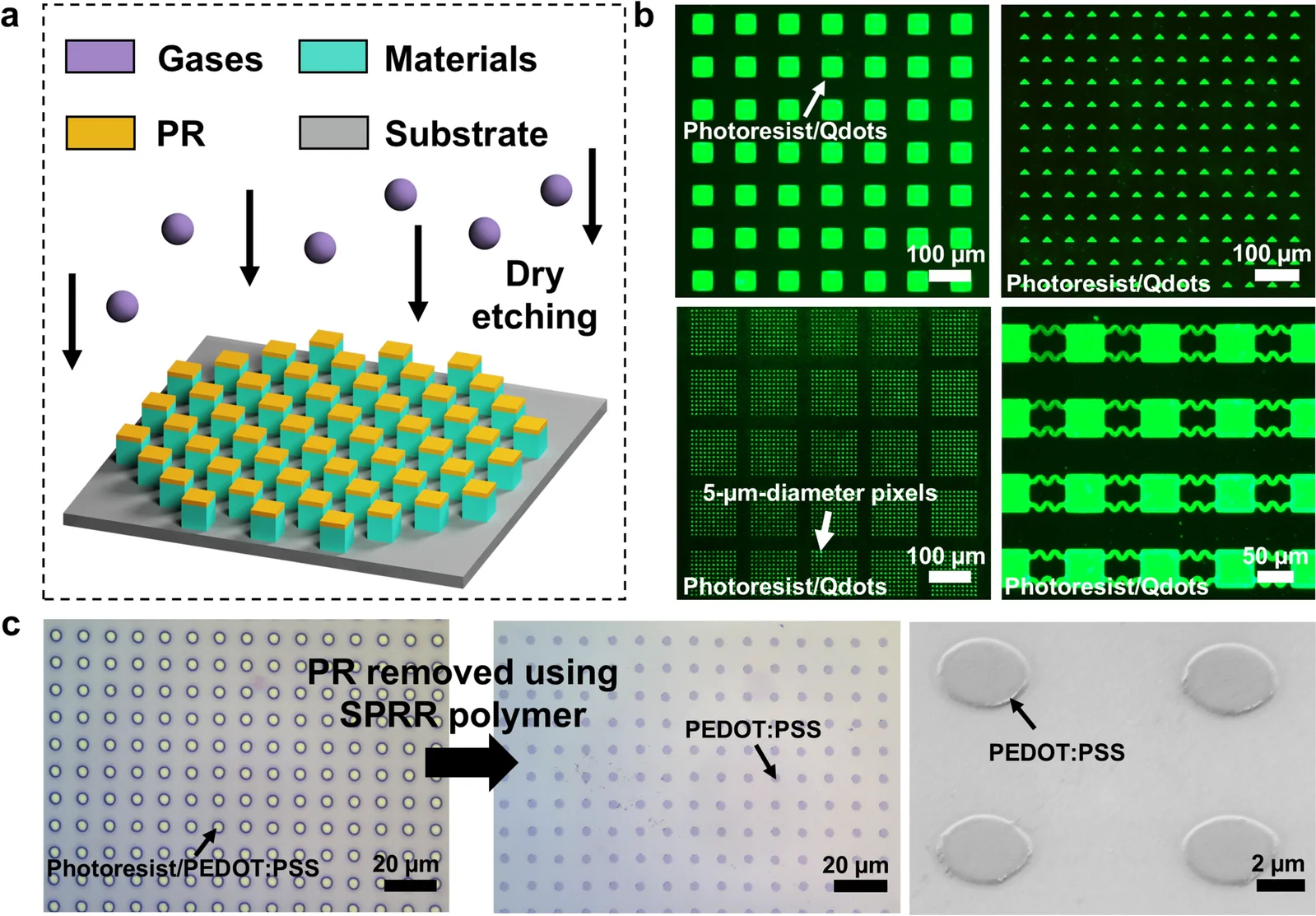
Susceptible material patterning via dry etching. **a** Schematic illustration of susceptible material patterning through photoresist transfer followed by dry etching. **b** Fluorescence images of patterned structures of SU-8 2002 photoresist/CsPbBr_3_ quantum dots on glass substrate, showing discrete and continuous patterns with feature size ranging from 5 to 50 µm. **c** Patterned PEDOT:PSS array on the glass substrate following photoresist transfer, dry etching, and photoresist removal using SPRR polymer

In this study, we also applied our photoresist transfer method to enable dry etching-based patterning on photolithography-incompatible materials, such as solvent-dispersed quantum dots and conductive polymers. These materials are typically susceptible to damage, swelling, or delamination during conventional photolithographic steps including resist coating, baking, or development [12, 39]. The process begins with coating a functional material layer onto the target substrate, followed by transferring a patterned photoresist layer onto the material surface. Conventional dry etching then defines the patterns with high fidelity, as illustrated in Fig. 4a.

Figure 4b shows patterned SU-8 2002/CsPbBr_3_ quantum dot layers on glass substrate after photoresist transfer and O_2_/Ar plasma etching, with feature sizes ranging from 5 to 50 µm, where the photoresist remained atop patterned qdots. The SEM images in Fig. S15 show the patterned features of photoresist/qdots, and Fig. S16 shows the height information of photoresist after dry etching. In the present configuration, the PR remains and mechanical removal of the photoresist may partially disturb the underlying quantum dot patterns, mainly due to the relatively weak adhesion between deposited quantum dots and the substrate surface. Achieving residue-free photoresist removal while preserving pattern integrity therefore requires further optimization of the quantum dot–substrate interface and process conditions. In the meantime, the current configuration remains relevant for several practical scenarios where the photoresist layer functions as a temporary structural or encapsulation component, such as rapid prototyping of color conversion layers or proof-of-concept optoelectronic devices, where precise material placement rather than resist-free exposure is the primary requirement. Figure 4c first shows the patterning result of SU-8 2002/PEDOT:PSS on glass substrate using the same approach. Following dry etching, the transferred photoresist can be selectively removed using the SPRR polymer carrier, creating discrete PEDOT:PSS array on the glass substrate. Overall, these demonstrations highlight the broad applicability and versatility of our transfer-based dry patterning approach, especially for materials previously considered incompatible with traditional lithographic techniques.

Details of material preparation and transfer procedures are provided in Experimental Section.

### A Sustainable “Dry Lift-Off” Strategy for Thin-Film Patterning

Recently, a two-step dry lift-off method was proposed for nanoscale Cr hard-mask patterning, in which sputtered Cr on patterned PMMA is mechanically removed using PDMS and PVA carriers, followed by O_2_ plasma etching to remove the remaining photoresist, demonstrating an effective carrier-assisted dry lift-off strategy for nanoscale hard-mask fabrication [40]. Different from this work, we directed toward scalable microfabrication scenarios, where the key challenge lies in achieving reliable and reusable removal of photoresist–metal stacks over large areas using conventional lithographic processes.

**Fig. 5:**
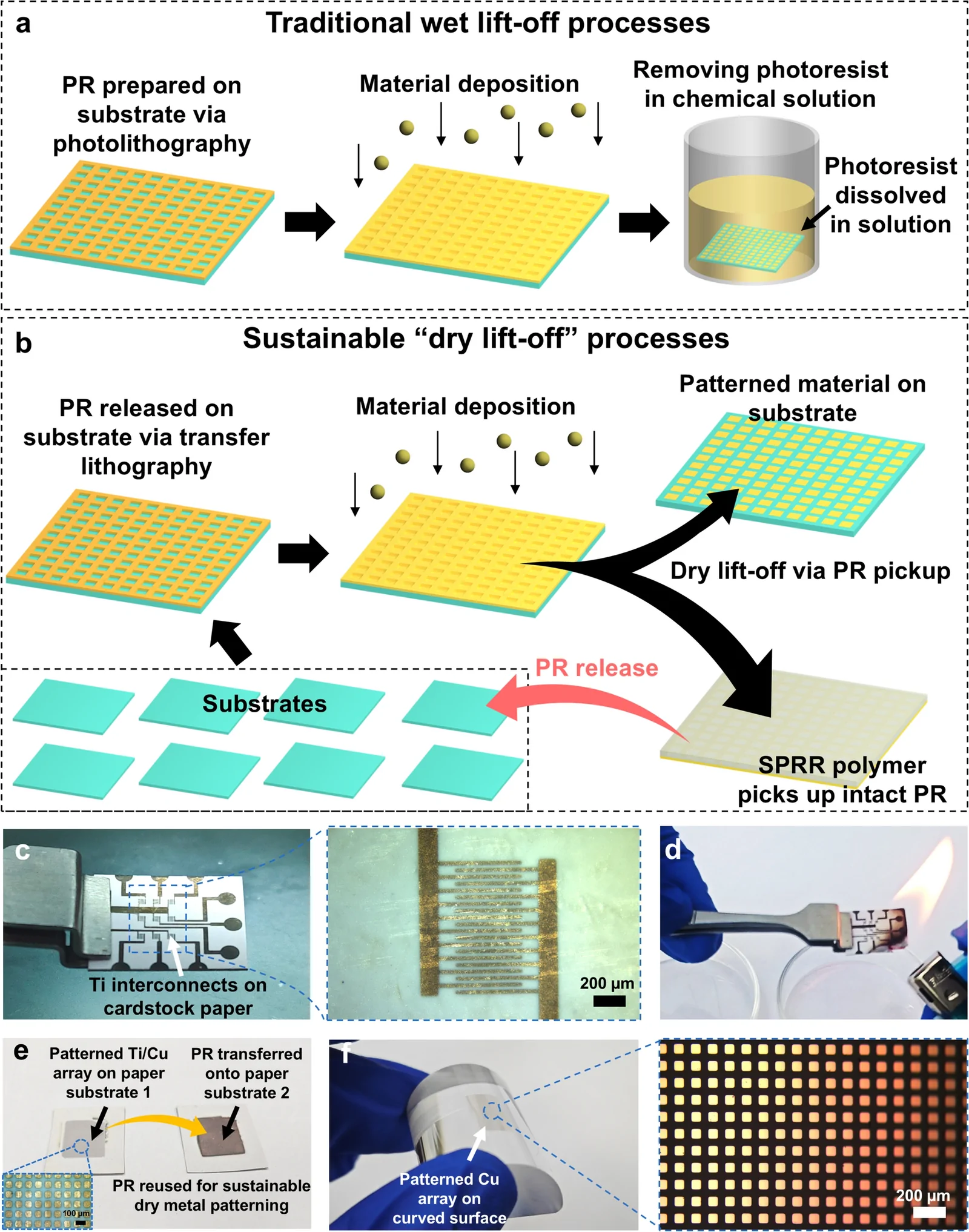
“Dry lift-off” processes for functional thin-film patterning on unconventional substrates. **a** Schematic illustration of traditional wet lift-off processes. **b** Schematic illustration of sustainable “dry lift-off” processes for batch patterning of functional thin film on unconventional substrates. **c** Optical images of a patterned interdigital electrode array of titanium (thickness: 100 nm) on a paper substrate using the “dry lift-off” process. **d** Rapid destruction of flammable paper-based electronics. **e** Demonstration of photoresist reuse: photoresist lifted from the metal-patterned paper substrate was re-released onto a second pristine paper substrate. **f** Copper array (thickness: 300 nm) patterned on a curved substrate using “dry lift-off” process

Building on the reversible photoresist transfer mechanism, we developed a sustainable “dry lift-off” process that enables scalable and rapid patterning of functional thin films. Unlike conventional lift-off methods (Fig. 5a**)** that rely on solvent dissolution to irreversibly remove photoresist, this approach avoids wet processing entirely. Moreover, it supports the reuse of both the photoresist and the SPRR polymer carrier and offers a sustainable alternative for batch microfabrication that is inaccessible with other transfer methods due to the potential challenges in integrality and fidelity of photoresist transfer.

As illustrated in Fig. 5b, the process begins with transferring photoresist onto the target substrate, followed by deposition of the desired thin film over the entire surface, including the photoresist. Following the established pickup protocol, the SPRR polymer carrier makes contact with the photoresist through the thin film that covers it, and then removes the underlying photoresist together with the film deposited on top. (The SEM images of photoresist/SPRR polymer after dry lift-off process are provided in Fig. S17.) As a result, only the film in the resist-free regions remains on the substrate, achieving dry thin-film patterning. In these processes, the thin film has good adhesion to photoresist, and thus, they can be considered as a whole structure, which can be firmly handled by the SPRR polymer carrier in dry lift-off processes via locking the thin-film coating of photoresist. Due to its reversible transition between rigid and rubbery states, the SPRR polymer allows the photoresist to remain intact on the carrier after lift-off, making it available for subsequent reuse. This enables a cyclic and solvent-free patterning route that eliminates the need for repeated photolithography and consuming photoresist in each cycle. To demonstrate this capability, we performed “dry lift-off” experiments using SU-8 2010 photoresist and titanium deposition on paper substrates, which is photolithography-inaccessible due to its solution-sensitive properties and remains challenges in high-resolution microfabrication. Figure 5c shows a titanium interdigital electrode array (thickness: 100 nm) patterned using this method. Compared with solution-degradable polymer-based transient electronics, paper-based electronics can be rapidly destructed by burning. Figure 5d demonstrates rapid destruction of fabricated paper-based electronics. Figure 5e further shows that the photoresist lifted from one substrate was successfully re-released onto a second substrate, where an identical patterning process was repeated, confirming the reuse of the same photoresist layer for a second cycle. To further evaluate the reusability of the photoresist, we performed multiple dry lift-off experiments, where both the photoresist and the transfer carrier were reused five times for solvent-free metal patterning on multiple glass substrates. As shown in Fig. S18, the consistently low pattern registration error across successive transfers, together with the structural integrity of the photoresist after repeated use, demonstrates the excellent sustainability of the SPRR polymer-based dry lift-off process. Maximum number of reuse cycles is determined by the process control of transfer lithography and subsequent microfabrication steps. For example, deposition methods with strong sidewall coverage (e.g., sputtering) may gradually modify the photoresist sidewall profile after repeated cycles, whereas directional deposition processes such as evaporation are less prone to sidewall accumulation and therefore minimize potential impacts on feature dimensions. By decoupling thin-film patterning from conventional photolithography and wet processing, the method substantially improves throughput and process sustainability and offers a promising route toward scalable, cost-effective microfabrication. “Dry lift-off” process was also used for patterning water-soluble substrates as shown in Fig. S19. Details of the “dry lift-off” protocol and experimental parameters are provided in Experimental Section.

### Transfer and “Dry Lift-Off” Enabled Functional Devices on Curved Surfaces

Curved electronics offer key advantages in optoelectronics and wearables, such as wide-angle imaging, efficient energy harvesting, and conformal sensing. However, in situ fabrication on non-planar geometries remains challenging due to the lack of effective methods for extending high-resolution photolithography from flat substrates to curved surfaces [10, 41]. To demonstrate the capability of our method in this context, we performed “dry lift-off” experiments on curved surface where a free-standing, 1-mm-thick SPRR polymer carrier was used. Figure 5f shows a copper thin film (thickness: 300 nm) patterned into a 50 µm × 50 µm square array on a half-cylinder substrate with a diameter of 2 cm.

**Fig. 6:**
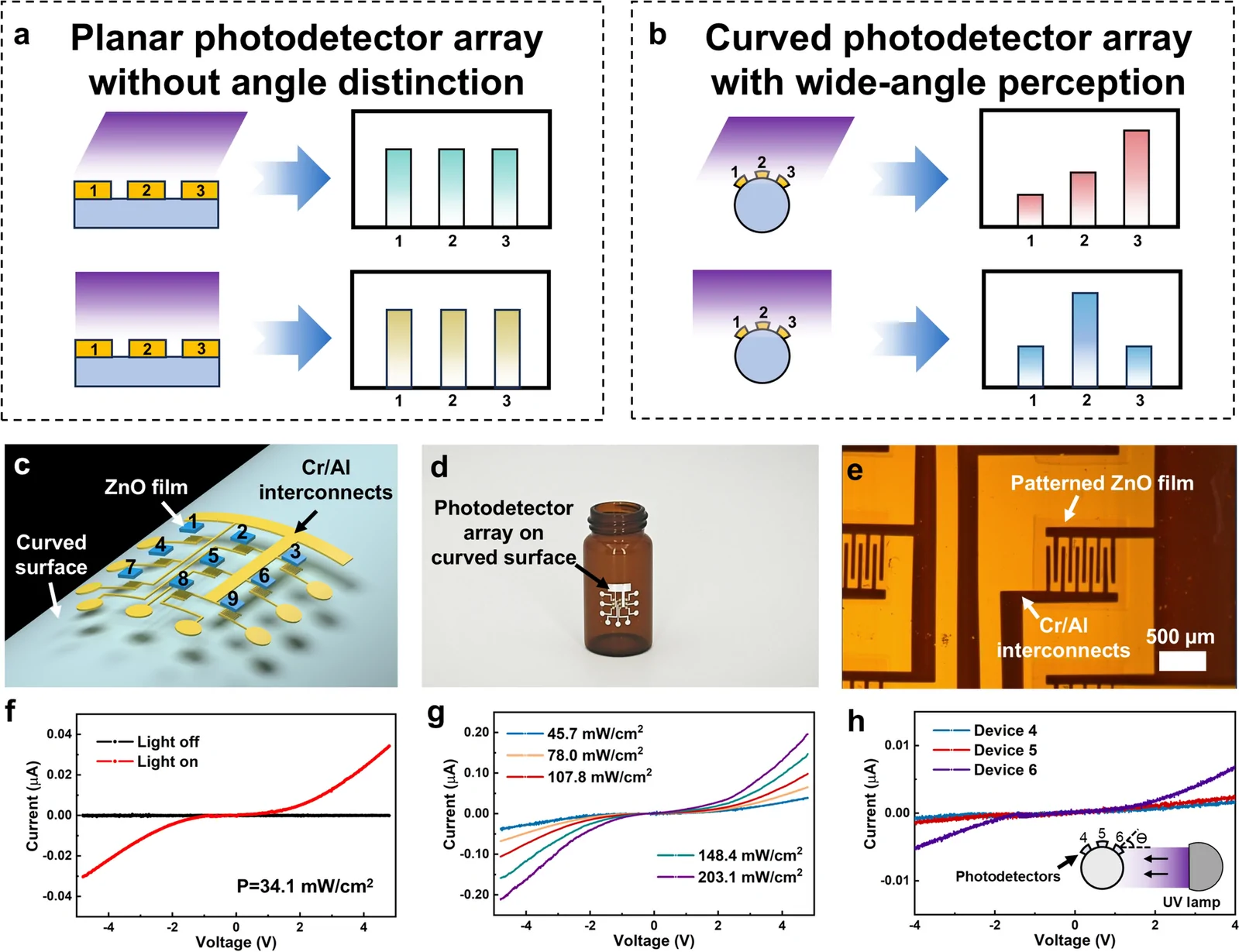
“Dry lift-off” enabled micro-sized UV photodetector array with wide-angle perception on a curved glass bottle. **a** Schematic illustration of planar photodetector array. **b** Schematic illustration of curved photodetector array with wide-angle perceptions. **c** Schematic of a 3 × 3 photodetector array (labeled 1–9) consisting of ZnO active layers and Cr/Al interconnects fabricated on a curved surface. **d—e** Optical images of photodetector array fabricated on the curved surface of a brown glass bottle. **f** I–V characteristics of the central photodetector (device 5) with and without 365—nm UV illumination (power intensity: 34.1 mW cm^−2^). **g** I–V response of device 5 under varying UV illumination power intensities. **h** I–V characteristics of photodetectors 4, 5, and 6 measured under angled side illumination. UV light was directed from the side of the curved bottle, resulting in varying incident angles across the detectors

As a further demonstration, we fabricated a 3 × 3 photodetector array on a curved surface. Compared with planar photodetector arrays (Fig. 6a), the curved array exhibit extra wide-angle perception capabilities (Fig. 6b). Figure 6c illustrates the device schematic, consisting of Cr/Al interconnects (20/250 nm thick) and photoactive ZnO layers (650 nm thick) arranged in a 3 × 3 array. Figure 6d, e shows optical images of the completed device directly fabricated on a brown glass bottle. To verify the functionality of the photodetector array, we characterized the performance of the photodetectors. First, we measured the response of the central photodetector (device 5) under top-down illumination, where the UV light was directed perpendicular to the center of the curved surface. As shown in Fig. 6f, under UV illumination at 365 nm with a power intensity of 34.1 mW cm^−2^ and a bias of 4 V, the photocurrent reached 0.0238 μA, while the dark current was 0.0001 μA, resulting in a relative current change ${{\left( {I_{{{\mathrm{light}}}} - I_{{{\mathrm{dark}}}} } \right)} \mathord{\left/ {\vphantom {{\left( {I_{{{\mathrm{light}}}} - I_{{{\mathrm{dark}}}} } \right)} {I_{{{\mathrm{dark}}}} }}} \right. \kern-0pt} {I_{{{\mathrm{dark}}}} }}$of 237. As the illumination intensity increased, the photocurrent rose accordingly, reaching 0.1307 μA (bias: 4 V) at 203.1 mW cm^−2^ (Fig. 6g), confirming stable and responsive photodetection behavior.

To demonstrate the wide-angle sensing capability of the photodetector array on a curved surface, we measured the I–V characteristics of three adjacent photodetectors (devices 4 to 6) under side illumination with 365-nm UV light, as shown in Fig. 6h. In this configuration, the UV light was directed toward the side of the glass bottle, perpendicular to its longitudinal axis, with a power intensity of 23.9 mW cm^−2^. Due to the curved geometry, the light struck each detector at a different angle. The central device (device 5) received light at a shallow, near-tangential angle, while device 6 received light more directly. As a result, device 6 exhibited a higher photocurrent, confirming that the array can resolve differences in incident light direction across the curved surface.

To assess device stability under practical mechanical disturbances, vibration reliability tests were performed. During the test, a magnetic stirring bar was placed inside the bottle to introduce continuous impact and vibration and the corresponding I–V characteristics before and after testing were recorded. As shown in Fig. S20, the nearly unchanged I–V responses confirm the robust mechanical stability of the devices fabricated directly on curved rigid surfaces.

Additionally, photodetectors with identical pattern designs were fabricated on planar glass substrates using standard methods. As shown in Fig. S21, comparative measurements between planar and curved devices were carried out and the extracted response times show comparable device dynamics, indicating that fabrication on curved substrates does not introduce observable degradation in response speed of device. The electrical characterization of the planar photodetectors under varying UV illumination intensities is also provided in Fig. S22.

Details of the fabrication processes for metal patterning and photodetector integration on curved substrates are provided in Experimental Section.

## Conclusions

We introduce a high-fidelity and scalable photoresist transfer method employing a sharp phase-changing rigid-to-rubbery polymer carrier that has a nearly 2300-fold change in storage modulus across a moderate melting temperature. During rigid-to-rubbery transition, the phase-changing polymer exists significantly adhesion-switchable capability enabling the transfer of photoresist from PDMS-coated donor wafer onto previously inaccessible substrates. Utilizing controlled transition between rigid, dimensionally stable state and soft, conformable state during alignment, pickup and release steps, this transfer method enables a wafer-scale (~ 4-inch) photoresist transfer with global registration error below 60 µm.

Unlike previously reported photoresist transfer method, our approach demonstrates general applicability for multiple commercial photoresists that are ready to use and not reliant on additional treatment. These photoresists, defined through standard photolithography, were successfully transferred onto a broad range of substrates previously incompatible with lithography, including flexible film, solvent-sensitive layers, curved and microtextured surfaces, and delicate materials. These transfer experiments highlight the generality and scalability of our method for broader adoption.

Combined with dry etching, we demonstrate a novel solvent-free method for patterning solvent-susceptible materials. It achieves high-resolution and multiscale patterning of quantum dots and organic semiconductors, with feature sizes down to 5 µm.

Additionally, we introduce a sustainable “dry lift-off” strategy for patterning functional thin film, such as semiconductors and metals. Different from tape-assisted transfer method, our transfer carrier and photoresist are both reusable for batch thin-film patterning, providing a new class for sustainable and scalable microfabrication. To demonstrate the transformative potential of this technology, we additionally fabricated a 3 × 3 micro-sized UV photodetector array with wide-angle sensing ability directly on a curved glass bottle.

This study systematically demonstrates the application potential of the photoresist transfer in various scenarios that are inaccessible to conventional lithography, opening new opportunities for groundbreaking applications of high-resolution microfabrication in emerging fields, such as flexible electronics, paper-based electronics, curved electronics, transient electronics, and optoelectronics.

Although the present work has demonstrated the broad application potential of wafer-scale transfer lithography for high-precision patterning on unconventional surfaces, several key limitations merit further clarification for future improvement. The minimum feature size demonstrated in this work is primarily determined by the lithographic definition on the donor substrate rather than by an intrinsic limitation of the transfer step. Achieving sub-micron features on compliant substrates such as PDMS typically requires more stringent optimization of resist thickness, exposure, and development compared with lithography on rigid silicon wafers. In principle, the transfer process is expected to preserve donor-defined resolution when pattern integrity is maintained. Extension toward near-micron features could be further explored through continued lithographic and process optimization. Specifically, further optimization of transfer registration accuracy and pattern resolution is required for large-area sub-micron patterning and multilayer device fabrication. Systematic improvements in the overall flatness, uniformity and surface quality of the transfer carriers, photoresist layers, and target substrates are critical for enhancing pattern fidelity during photolithography and transfer processes. Additionally, extending the transfer lithography process to thinner commercial photoresists (e.g., PMMA) would enable higher-resolution patterning.

## Supplementary Information

Below is the link to the electronic supplementary material.

Supplementary file1 (DOCX 43566 KB)
