# Supplementary material for: Scalable and Sustainable Dry Microfabrication Enabled by High-Precision and Wafer-Scale Transfer Lithography of Commercial Photoresists
Source: Nanomicro Lett. 2026 May 7;18:357. doi: 10.1007/s40820-026-02215-7 (PMC13149809; doi:10.1007/s40820-026-02215-7)
Supplement: Supplementary file 1 — Supplementary file1 (DOCX 43566 KB) [file 40820_2026_2215_MOESM1_ESM.docx]

Supporting Information for

**Scalable and Sustainable Dry Microfabrication Enabled by High-Precision and Wafer-Scale Transfer Lithography of Commercial Photoresists**

Qinhua Guo^1^, Zhiqing Xu^1^, Lizhou Yang^1^, Jingyang Zhang^1^, Yawen Gan^1^, Jiajun Zhang^1^, Jiahao Jiang^1^ and Yunda Wang^1, 2^, *

^1^ Smart Manufacturing Thrust, The Hong Kong University of Science and Technology (Guangzhou), Guangzhou 511400, P. R. China

^2^ Department of Mechanical and Aerospace Engineering, The Hong Kong University of Science and Technology, Hong Kong SAR 999077, P. R. China

*Corresponding author. E-mail: [ydwang@ust.hk](mailto:ydwang@ust.hk) (Yunda Wang)

**S1 Material**

Polydimethylsiloxane (SYLGARD 184) was purchased from Dow Corning, America. n-Hexane was obtained from Macklin Biochemical Technology Co., Ltd, China. Commercial free-standing films including PI, PET and PU, were cleaned as flexible receiver substrates without undergoing additional treatment. Silicone gel-film (6.5 mil PF-X4 gel-film) were purchased from Gel-Pak, America. PVA powder (degree of hydrolysis: 87~89 mol%, viscosity: 54-66 mPa) was obtained from Macklin Biochemical Technology Co., Ltd, China. Fluoropolymer solution (FW-8800) was purchased from Dongguan Fuwang New Material Co., Ltd. CsPbBr_3_ qdots solution (concentration: 50 mg ml^-1^) was purchased from Gamudot Advanced Materials Co., Ltd. CsPbBr_3_ qdots solution (concentration: 5 mg ml^-1^) was obtained from Guangzhou Periodic Table biomaterials Co., Ltd. 1H,1H,2H,2H-Perfluorooctyltrichlorosilane (FOTS) was purchased from Macklin Biochemical Technology Co., Ltd, China. The AZ5214E photoresist was purchased from AZ Electronic Materials, America. SU-8 2002 and SU-8 2010 were obtained from MicroChem, America. AR 300-80 new was acquired from Allresist, Germany.

**S2 Preparation of SPRR polymer carriers**

**S2.1 SPRR polymer/glass carrier**

First, a layer of adhesion promoter (3M 94 Primer) is applied onto a cleaned glass substrate. Subsequently, the liquid SPRR pre-polymer is dispensed in the gap between the glass substrate and a FOTS-treated glass substrate which are separated by 1-mm-thick spacers. The pre-polymer is then cured using 365 nm UV light at 300 mW cm^-^² for 3 min, followed by baking at 80 ℃ for 2 hours. Finally, the SPRR polymer/glass carrier is obtained by removing the glass cover at 80 ℃.

**S2.2 Free-standing SPRR polymer**

First, the SPRR pre-polymer is dispensed into the gap between two FOTS-treated glass substrates which are separated by a 1-mm-thick spacer. Then, the SPRR pre-polymer is cured using 365 nm UV light at 300 mW cm^-^² for 3 min, followed by baking at 80 ℃ for 2 hours. Finally, the free-standing SPRR polymer is obtained by removing two glass substrates at 80 ℃.

**S3 Preparation of PDMS-coated wafer**

**S3.1 15-µm-thick PDMS coating on silicon wafer**

First, a PDMS prepolymer is diluted by mixing the base solution and the curing agent into n-hexane solution at a weight ratio of 10:1:10 (base solution: curing agent: n-hexane). This diluted PDMS prepolymer is then spin-coated onto a cleaned silicon wafer at 1000 rpm for 30 s. Finally, the PDMS/silicon substrate is obtained by curing PDMS coating at 80℃ for 3 hours.

**S3.2 110-µm-thick PDMS coating on glass wafer**

First, a PDMS prepolymer is prepared by mixing base solution and curing agent at a weight ratio of 10:1. This PDMS prepolymer is then spin-coated on a cleaned glass substrate at 1000 rpm for 30 s. Finally, the PDMS/glass substrate is obtained by curing it at 80 ℃ for 3 hours.

**S4 Photoresist preparation on PDMS-coated rigid wafer**

**S4.1 Discrete SU-8 2002 photoresist structures**

First, the PDMS/silicon wafer undergoes oxygen plasma treatment (Tergeo-Plus, PIE Scientific) at 50 W for 1 min to enhance surface wettability. Next, an adhesion promoter (AR 300-80 new) is applied to the wafer via spin-coating at 4000 rpm for 30s, followed by a baking at 180 ℃ for 2 min. Subsequently, a layer of SU-8 2002 photoresist is spin-coated onto the PDMS/silicon wafer at 4000 rpm for 30 s. Subsequently, the photoresist is baked at 65 ℃ for 2 min and then at 95 ℃ for 5 min. Following this, the photoresist is exposed to 365 nm UV light (URE-2000S/AL) at an intensity of 9 mW cm^-^² for 11 s, followed by a post-exposure baking at 65 ℃ for 2 min and then at 95 ℃ for 5 min. Finally, the SU-2002 photoresist is developed to create patterned structures on the PDMS/silicon wafer.

**S4.2 Discrete SU-8 2010 photoresist structures**

First, the oxygen plasma treatment (Tergeo-Plus, PIE Scientific) is performed on the PDMS/glass wafer surface at 50 W for 60 s. Following this, an adhesion promoter (AR 300-80 new) is spin-coated on the PDMS/glass wafer at 4000 rpm for 30 s and then baked at 180 ℃ for 2 min. Next, a SU-8 2010 photoresist layer is spin-coated on PDMS/glass wafer at 3000 rpm for 30 s, followed by soft baking at 65 ℃ for 1 min and then at 95 ℃ for 4 min. The photoresist is then exposed to 365 nm UV light (URE-2000S/AL) at an intensity of 9 mW cm^-^² for 25 s. Following this, the substrate undergoes post-exposure baking at 65 ℃ for 1min and then at 95 ℃ for 4 min. Finally, the photoresist is developed to obtain discrete patterns on PDMS/glass substrate.

**S4.3 Continuous SU-8 2010 photoresist film**

First, a SU-8 2010 photoresist layer is spin-coated onto PDMS/glass wafer at 3000 rpm for 30 s. This is followed by a soft-baking process at 45 ℃ for 1 h. The baked photoresist is then exposed to 365 nm UV light (URE-2000S/AL, intensity: 9 mW cm^-^², duration: 10 s), followed by post-exposure baking at 65 ℃ for 1min and then at 95 ℃ for 4 min. Finally, the photoresist is developed to obtain continuous patterned photoresist film on PDMS/glass substrate.

**S4.4 Discrete AZ5214E photoresist structures**

First, the PDMS/silicon surface undergoes an oxygen plasma treatment (LEBO Science, PT500) at 100 W for 60 s to improve its surface wettability. Following this, an adhesion promoter (AR 300-80 new) is spin-coated on the PDMS/silicon wafer at 4000 rpm for 30 s and subsequently baked at 180 ℃ for 2 min. Next, an AZ5214E photoresist layer is spin-coated on the PDMS/silicon substrate at 3000 rpm for 30 s and then baked at 100 ℃ for 100 s. Finally, the photoresist is exposed to 365 nm UV light (SUSS MA/BA6 Gen4) at 15 mW cm^-^² for 6 s and then developed to create patterned photoresist structures on the PDMS/silicon substrate.

**S5 Preparation of receiver substrates**

**S5.1 PVA film**

First, PVA powder (2 grams) is dissolved in deionized water (40 ml) with stirring. Then, the PVA solution is poured into a cleaned plastic petri dish. The PVA solution is then naturally dried at room temperature. Finally, the PVA film is peeled off from the petri dish to obtain a water-solvable receiver substrate.

**S5.2 Fluoropolymer film**

First, a solution (FW-8800) of fluoropolymer is poured into a cleaned plastic petri dish. Then, the fluoropolymer is naturally dried and solidified in room temperature. Following this, the fluoropolymer film is obtained by removing the plastic petri dish.

**S5.3 CsPbBr_3_ film on glass substrate**

First, a glass substrate undergoes oxygen plasma treatment (Tergeo-Plus, PIE Scientific) at 50 W for 60 s. Then, a CsPbBr_3_ solution (concentration: 5 mg ml^-1^) is spin-coated on the glass substrate at 1000 rpm for 30 s, followed by baking at 80 ℃ for 15 min. The spin-coating and baking processes are performed in the nitrogen atmosphere. The prepared CsPbBr_3_ film will be a receiver substrate of AZ5214E photoresist. (See in Fig. 3h).

**S5.4 SU-8 3025 structures fabricated on PDMS**

First, a PDMS coated glass substrate undergoes oxygen plasma treatment (Tergeo-Plus, PIE Scientific) at 50 W for 60 s to improve its surface wettability. Then, an adhesion promoter (AR 300-80 new) is spin-coated on PDMS/glass substrate at 4000 rpm for 30 s, followed by a baking at 180 ℃ for 120 s. SU-8 3025 photoresist layer is then spin-coated on the PDMS substrate at 3000 rpm for 30 s. The photoresist layer is then baked at 65 ℃ for 1min and then at 95 ℃ for 10 min. Subsequently, the photoresist layer is exposed to 365 nm UV light (URE-2000S/AL) at a power density of 9 mW cm^-^² for 25 s. Following this, the substrate is baked at 65 ℃ for 1min and then at 95 ℃ for 10 min. Finally, the photoresist is developed to create pattern on the PDMS/glass substrate.

**S5.5 SU-8 2010 photoresist layer with sandpaper-replicated protrusions**

First, a PDMS prepolymer (base solution: curing agent=10:1) is poured onto a sandpaper (P800, grain size: 21.8 µm), and then cured at 80 ℃ for 3 hours. Subsequently, a PDMS mold is obtained by removing the sandpaper. Then, the SU-8 2010 photoresist is dispensed into a gap between a clean glass substrate and the PDMS mold, which are separated by spacers. The SU-8 2010 photoresist is then cured at 65 ℃ for 30 min. Then, the SU-8 2010 photoresist is exposed to 365-nm UV light at a power density of 9 mW cm^-^² for 1 min. After performing baking at 65 ℃ for 1 min and 95 ℃ for 10 min, PDMS mold is removed to obtain SU-8 2010 photoresist/glass substrate, which has sandpaper-replicated protrusions on its surface.

**S6 Transfer of commercial photoresist onto diverse unconventional substrates**

In experiments involving the transfer of photoresists onto diverse unconventional substrates, the pickup and release steps were manually performed. Based on a transfer area of L × L and a duration over T, the average fracture propagation speed is estimated to be below L/T.

**S6.1 High-precision, wafer-scale photoresist transfer**

First, a 4-inch SPRR polymer/PET carrier is laminated onto 4-inch PDMS/silicon donor substrate using a commercial hot laminator (Tongrong Office Equipment, FGK 330-6R) at room temperature. Subsequently, the laminated components undergo a secondary lamination process with the laminator set to a heating temperature of 80 °C. After cooling down to room temperature, the photoresist structures can be picked up by separating the SPRR polymer/PET carrier from the donor substrate at an estimated average fracture propagation speed less than 2 mm s^-1^. Before the photoresist release, a free-standing PVA film with a thickness of 100 µm is heatedly laminated on a cleaned 4-inch silicon wafer using the laminator. Similarly, the SPRR polymer/PET carrier carrying photoresist is then laminated on the PVA/silicon substrate at room temperature, followed by a secondary lamination at 80 ℃. For the photoresist release, both the SPRR polymer/PET carrier and the PVA/silicon substrate are heated to 80 ℃ on a hot plate. The SPRR polymer/PET carrier is then peeled away from the PVA/silicon substrate at an average fracture propagation speed estimated to be less than 2 mm s^-1^. Finally, the free-standing PVA film with wafer-scale discrete patterns is obtained by separating it from the silicon substrate. For AZ5214E photoresist transfer, Fots vapor treatment was performed on the photoresist before transfer to avoid the unwanted permeation between polymer under long-term heating.

**S6.2 Photoresist transferred onto flexible or solvent-incompatible substrates**

First, the SPRR polymer/glass carrier is brought into contact with the photoresist on the donor substrate at 80 ℃. After cooling down to room temperature, the photoresist is picked up by separating SPRR polymer/glass carrier from the donor substrate, with an estimated average fracture propagation speed of less than 2 mm s^-1^. For photoresist release, the SPRR polymer/glass carrier brings the photoresist into contact with receiver substrate at 80 ℃, and then is separated from the receiver substrates at an estimated average fracture propagation speed less than 2 mm s^-1^.

**S6.3 Photoresist transferred onto curved surfaces**

First, a 1-mm-thick free-standing SPRR polymer carrier contacts the photoresist on the donor substrate at 80 ℃. After cooling down to room temperature, the photoresist is picked up by separating the SPRR polymer carrier from the donor substrate at an estimated average fracture propagation speed less than 2 mm s^-1^. For photoresist release, the SPRR polymer brings photoresist into intimate contact with the curved surface at 80 ℃. After separating the rubbery SPRR polymer from the curved surface at an average fracture propagation speed estimated less than 2 mm s^-1^, the photoresist is released onto the curved surface.

**S6.4 Patterning on pre-structured 3D topographies**

The pickup step of photoresist transfer is manually performed. First, the SPRR polymer/glass carrier is brought into contact with the photoresist on the donor substrate at 80 ℃. After cooling down to room temperature, the photoresist is picked up by separating SPRR polymer from donor substrate with an average fracture propagation speed estimated to be below 2 mm s^-1^. Subsequently, a 6-axis transfer system is employed to precisely control the release process of the photoresist. The rigid SPRR polymer/glass carrier brings the photoresist into contact with the receiver substrate under control of the transfer system. Then, the SPRR polymer is consistently heated by a heat gun set at 110 ℃ to maintain in rubbery state. Finally, the SPRR polymer in rubbery state is retracted at a speed of 5 µm s^-1^ to release photoresist onto pre-structured substrates.

**S7 Processes of susceptible material patterning**

**S7.1 CsPbBr_3_ qdots patterning**

The process begins with oxygen plasma treatment on a smooth glass substrate to enhance its surface wettability. Then, a CsPbBr_3_ quantum dot solution (concentration: 50 mg ml^-1^) is spin-coated on the cleaned glass substrate at 1000 rpm for 30 s, followed by baking at 80 ℃ for 15 min. The spin-coating and baking processes are all performed in a nitrogen atmosphere. Subsequently, a patterned SU-8 2002 photoresist layer is transferred from PDMS/silicon donor substrate onto CsPbBr_3_ layer using SPRR polymer/PET carrier following the established pickup and release protocols. Then, the substrate undergoes a dry etching process in the plasma cleaner chamber (ASTRO PACTO -10H) at a power of 600 W with O_2_/Ar gas mixture (flow rate: 100/100 sccm, pressure: 8.5 Pa) to create patterned photoresist/CsPbBr_3_ quantum dot structures on the glass substrate.

**S7.2 PEDOT: PSS patterning**

First, a cleaned glass substrate undergoes oxygen plasma treatment to improve its surface wettability. Then, a PEDOT:PSS solution (OE-000, concentration: 1.1%) is spin-coated on the substrate at 3000 rpm for 30 s, followed by baking at 120 ℃ for 5 min. Subsequently, the SU-8 2002 photoresist is transferred onto PEDOT:PSS film using a SPRR polymer/PET carrier. Then, the substrate is etched in a plasma cleaner chamber (CIF) at a power of 300 W with air (pressure: 35 Pa) to obtain discrete photoresist/PEDOT:PSS structures. Finally, the photoresist is removed using SPRR polymer/glass carrier following the pickup protocol, creating patterned PEDOT:PSS polymer on the glass substrate.

**S8 Sustainable dry lift-off processes**

**S8.1 Patterned metal film on paper substrate**

First, a SU-8 2010 photoresist film is patterned on the donor substrate. Then, the photoresist is transferred onto cardstock paper substrate using SPRR polymer/glass carrier. The heating temperature is set as 60 ℃. Subsequently, specific metal material is sputtered onto paper substrate. Then, the SPRR polymer is heated to rubbery state and gently pressed against the target substrate to establish intimate contact with the top of photoresist (now covered by the deposited film). In these processes, the SPRR polymer can maintain isolation to the sputtered materials on target substrates, minimizing the contact damage on the sputtered materials. Finally, the photoresist is picked up by the rigid SPRR polymer/glass carrier following the established pickup protocol.

**S8.2 Patterned Ti film on PVA substrate**

First, a continuous patterned film of SU-8 2010 photoresist is prepared on the donor substrate. Then, the photoresist is transferred onto PVA substrate using SPRR polymer/glass carrier. Subsequently, Ti with specific thickness is sputtered onto PVA substrate. Finally, the photoresist is picked up by the SPRR polymer/glass carrier following the established pickup protocol.

**S8.3 Patterned Cu film on curved surfaces**

First, a patterned SU-8 2010 photoresist layer is prepared on the donor substrate. Then, the photoresist is transferred onto curved surface using the free-standing SPRR polymer carrier, following the photoresist transfer protocols. The curved surface is then sputtered with 300-nm-thick copper film. Finally, the photoresist is picked up by the SPRR polymer following the pickup protocol, creating discrete copper film on the curved surface.

**S8.4 Fabrication of photodetector array on glass bottle**

First, a SU-8 2010 photoresist layer is prepared on donor substrate through photolithography and then is transferred onto a brown glass bottle. Following this, Cr/Al metal layers (thickness: 20/250 nm) are sputtered onto the curved surface of the glass bottle. Then, the photoresist is then mechanically lifted using the free-standing SPRR polymer, creating Cr/Al metal interconnects on the glass bottle. Subsequently, a patterned SU-8 2010 photoresist layer, designed for ZnO film, is transferred onto the glass bottle with the free-standing SPRR polymer, where the patterns of photoresist are aligned with Cr/Al electrode pattern under a microscope. Then, a 650-nm-thick ZnO layer is sputtered onto curved surface. Finally, the photoresist is removed using the freestanding SPRR polymer, completing photodetector array fabrication on the glass bottle.

**S9 Supplementary Text**

**Section S1 Working principle of the SPRR polymer-based photoresist transfer method**

The adhesion-switching behavior of SPRR polymer relies on the huge modulus difference between rigid and rubbery states, where the melting-crystallization transition of the SA crystalline aggregates in the SPRR polymer significantly determines the huge storage modulus change range over a narrow phase change temperature range. The long-chain UDA is used as the polymer framework to improve the toughness of the SPRR polymer in the rubbery state. In rigid state, the SA crystalline aggregates act as hard segments to maintain the high storage modulus of SPRR polymer. When the temperature of SPRR polymer exceeds the melting temperature, SA crystalline aggregates rapidly melt and become plasticizers to soften the polymer, resulting in a rapid decline of storage modulus over a narrow temperature range. As shown in Fig. 1a, increasing the SA weight fraction in the SA-UDA copolymer facilitates SPRR polymer with higher modulus in rigid state and lower value in rubbery state, demonstrating a nearly 2300-fold change in storage modulus of SPRR polymer (80 parts SA in 100 parts SA-UDA copolymer) over a narrow temperature range.

During the pickup phase, the SPRR polymer carrier has stable conformal contact with the photoresist after contact-heating-cooling processes. In this state, the SPRR polymer carrier and photoresist is rigid and elastic, while the PDMS is viscoelastic. The critical energy release rate for rigid SPRR polymer/photoresist interface ($G_{crit}^{Rigid\text{ }SPRR\text{ }polymer/photoresist}$) can be considered as an inherent material property of the interface and is independent to the separation speed of the SPRR polymer carrier. By contrast, the critical energy release rate $G_{crit}^{photoresist/PDMS}(v)$ of photoresist/PDMS interface monotonically increase with the separation speed [42]:

$G_{crit}^{photoresist/PDMS}(v)\text{=}G_{0}[1+(\frac{v}{v_{0}})^{n}]$ **(S1)**

where $G_{0}$ is the critical energy release rate when the separation velocity $v$ approaches zero, $v_{0}$ is a reference separation speed at which the critical energy release rate equal to 2$G_{0}$. The power-law exponent n primarily represents the rate sensitivity of viscoelastic energy dissipation of materials (PDMS and rubbery SPRR polymer) at the crack tip. It is a material- and temperature-dependent parameter that governs how steeply the critical energy release rate increases with peeling speed. Materials with stronger viscoelastic properties exhibit larger values of n, leading to a steeper rise in the critical energy release rate with increasing separation velocity. In addition, as temperature increases, the viscoelastic dissipation weakens, resulting in a decrease in n. According to the reported classical fracture competition theory [42], the principle for photoresist pickup can be considered as the fracture competition between SPRR polymer/photoresist interface and photoresist/PDMS interface, where the fracture propagation occurs at the interface that has lower critical energy release rate. When the SPRR polymer carrier is retracted at a speed lower than a critical value, the critical energy release rate of photoresist/PDMS interface is reduced and lower than that of SPRR polymer/photoresist interface ($G_{crit}^{Rigid SPRR\text{ }polymer/photoresist}>G_{crit}^{photoresist/PDMS}(v)$), and thus the facture propagation preferentially occurs at the photoresist/PDMS interface.

During photoresist release phase, the SPRR polymer brings photoresist into contact with the elastic receiver substrates in rubbery state. In this state, the rubbery SPRR polymer exhibits viscoelastic behavior. The critical energy release rate of SPRR polymer/photoresist interface monotonically increases with separation speed of SPRR polymer, as follows:

**(S2)**

Where the $G_{1}$ is the critical energy release rate of rubbery SPRR polymer/photoresist interface when the separation velocity $v$ of SPRR polymer near to zero, $v_{1}$ is a reference separation speed at which the critical energy release rate double to $G_{1}$, and is a parameter determined from the experiments. On the contrary, the critical energy release rate of the photoresist/substrate interface can be considered as a value independent to separation speed. Thus, when the rubbery SPRR polymer carrier is separated from the receiver substrate at a speed lower than a critical value, the critical energy release rate of rubbery SPRR polymer/photoresist is reduced and lower than that of the photoresist/substrate interface ($G_{crit}^{Rubbery SPRR\text{ }polymer/photoresist}(v)<G_{crit}^{photoresist/substrate}$), enabling the fracture propagation occurred at rubbery SPRR polymer/photoresist interface. Actually, properties of the receiver substrate play a critical role in determining the fracture interface during the release step. Differences in surface energy, stiffness, and intrinsic adhesion to the photoresist largely influence the critical energy release rate at the photoresist/receiver interface. For successful release, the critical energy release rate of the photoresist/receiver interface must be higher than that of the rubbery SPRR polymer/photoresist interface; otherwise, the photoresist would remain adhered to the SPRR polymer rather than being released onto receiver substrate. Under the experimental conditions of this study, the rubbery SPRR polymer is carefully formulated to exhibit sufficiently weak adhesion to photoresists, allowing reliable release across a wide range of receiver substrates. Under certain extreme conditions, such as highly anti-adhesive receiver materials or strongly microtextured surfaces, the release process may become more challenging, and additional surface or process optimization may be required to ensure reliable transfer.

In addition to the dramatic modulus switching of the SPRR polymer, the interfacial energy between the SPRR polymer and the photoresist plays a critical role in the reversible adhesion switching process. The reversible adhesion switching behavior is governed by the combined effects of modulus-dependent contact mechanics and the intrinsic interfacial energy (work of adhesion) between the SPRR polymer and the photoresist. Excessively high interfacial energy may lead to irreversible bonding and hinder release, whereas insufficient interfacial adhesion can compromise pickup reliability.

**Section S2 Interface adhesion research**

To analyze adhesion transition behaviors of SPRR polymer and interface competition in lithography transfer, we performed adhesion characterization for different interfaces via 90-degree tape peeling tests. We utilized a setup equipped with a universal testing machine, a 90° peel test fixture, load cells and a hot plate. The hot plate was employed to regulate the temperature of samples. The 90° peel test fixture and universal testing machine were linked together to ensure synchronization of lateral displacement of tested sample with the vertical peeling length of tape, as the tape was peeled vertically from one end at a steady speed$v$. Load cells were utilized to measure the vertical force $F$ perpendicular to separated interfaces. Thus, the energy release rate can be expressed as:

$G=\frac{F}{w}$ **(S3)**

where the $w$ is the width of crack propagation.

As shown in Fig. S4a, to evaluate the energy release rate of rubbery SPRR polymer/photoresist interface, we conducted multiple 90-degree tape peeling tests on SU-8 2010 photoresist/rubbery SPRR polymer block at 80 ℃. Before performing the peeling tests, the 20-mm-width photoresists were initially laminated onto 1-mm-thick SPRR polymer blocks (with a supportive glass substrate), following photoresist pickup protocol. Figure S4b demonstrates the variation in measured adhesion relative to fracture propagation distance across different peeling speeds. The speed-dependent energy release rate of rubbery SPRR polymer/photoresist interface during steady fracture propagation can be further obtained as shown in Fig. S4c, demonstrating a minimized value of 0.7 J m^-2^ at a low peeling speed of 0.2 mm s^-1^. The speed-dependent energy release rate function of rubbery SPRR polymer/photoresist interface can also be determined as:

$G_{crit}^{Rubbery\text{ }SPRR\text{ }polymer/photoresist}(v)=0.59[1+(\frac{v}{1.146})^{0.92}]$ (S**4**)

Furthermore, to evaluate the adhesion stability of the SPRR polymer, repeated 90° peeling tests on a single SPRR polymer block were conducted (Fig. S5), demonstrating only minor variation in adhesion performance over multiple cycles.

Figure S6a demonstrates 90-degree tape peeling tests on SU-8 2010 photoresist film that is firmly locked by rigid SPRR polymer/glass substrate. Figure S6b shows the energy release rate measurement of tape/photoresist interface when the separation occurs at the tape/photoresist interface. Figure 6c shows the energy release rate of tape/photoresist interface during steady fracture propagation demonstrating a high value of 77.8 J m^-2^ at a peeling speed of 4 mm s^-1^. The speed-dependent adhesion of tape/photoresist interface also can be expressed as:

$G_{crit}^{Tape/Photoresist}(v)=20.09[1+(\frac{v}{0.33})^{0.44}]$ **(S5)**

When the tape was peeled at speeds exceeding 5 mm s^-1^, separation occurred at the rigid SPRR polymer/photoresist interface. Therefore, our analysis focuses on scenarios where the tape peeling speed is lower than 4 mm s^-1^. According to fracture competition mechanism, the energy release rate of rigid SPRR polymer/photoresist interface is higher than that of tape/photoresist interface when the fracture propagation occurs at the latter. Thus, the measured energy release rate of the tape/photoresist interface can be used to estimate the adhesion strength range of rigid SPRR polymer/photoresist interface. Combined these findings with adhesion measurement of rubbery SPRR polymer/photoresist interface, the adhesion switching ratio of pickup to release for non-structured, flat-surface photoresist film can be inferred higher than 110:1. Direct measurement of velocity-dependent energy release rate at rigid SPRR polymer/photoresist interface using conventional 90° peeling configurations is challenging. In the rigid state, the interface is dominated by elastic response with minimal viscoelastic dissipation, such that crack propagation becomes unstable once initiated. Under these conditions, the crack velocity is no longer governed by the externally imposed peeling speed, preventing reliable extraction of rate-dependent energy release rates using standard peeling analysis. Previous studies on analogous shape-memory polymer systems have therefore characterized rigid-state adhesion using pull-off force measurements rather than velocity-dependent peeling experiments, supporting the assumption of speed-independent interfacial behavior [43].

Figure S7a demonstrates the schematic of 90-degree tape peeling test on photoresist that was initially prepared on PDMS-coated silicon substrate. Figure S7b shows energy release rate measurements when the separation occurs at photoresist/PDMS interface under different peeling speeds. Figure S7c shows speed-dependent energy release rate of photoresist/PDMS interface during steady fracture propagation, with the function relationship expressed as:

$G_{crit}^{Photoresist/PDMS}(v)=1.691[1+(\frac{v}{0.488})^{0.678}]$ **(S6)**

Combined with Equation S4-S6, the interface fracture competition mechanism in transfer lithography can be schematically illustrated in Fig. 1f. The rigid SPRR polymer/photoresist interface can be considered as elastic system that is expected to exhibit speed-independent energy release rate.

Additionally, we performed 90° tape peeling experiments to evaluate the adhesion between the SU-8 2010 photoresist and the PVA receiver substrates. The SU-8 photoresists layers were first transferred onto the PVA receiver substrate using the SPRR polymer before the adhesion tests. Figure S8 shows the adhesion measurements for the tape/photoresist interfaces at 80 ℃. In these tests, the tape’s adhesion is insufficient due to the heating-induced attenuation and thus the photoresist can’t be peeled off even though the separation speed was accelerated to 8 mm s^-1^. Under these conditions, the critical energy release rate of photoresist/PVA substrate interface can be inferred to exceed the measured values for the tape/photoresist interface (G_PVA/Photoresist_>G_Tape/Photoresist_). Given the elastic nature of the receiver substrate, the critical energy release rate of photoresist/PVA substrate interface can be regarded as a speed-independent constant. Therefore, the G_PVA/Photoresist_ can be inferred to be higher than 21.04 J m^-2^. Figure S8c further compares the energy release rates of the tape/photoresist interface with that of the rubbery SPRR polymer/photoresist interface, thereby demonstrating the significant adhesion strength contrast between the rubbery SPRR polymer/photoresist interface and photoresist/PVA substrate interface. Overall, these results provide a more quantitative view of the photoresist release process.

We also conducted tape peeling tests on the photoresist/PVA substrate at room temperature. As shown in Fig. S9, when the tape was peeled at 0.2 mm s^-1^, the fracture occurred at the tape/photoresist interface, confirming that the critical energy release rate of photoresist/PVA substrate interface exceeds the measured tape/photoresist values (25.65 J m^-2^). When the peeling speed increased to 0.5 mm s^-1^, the photoresist layers were picked up from the PVA receiver substrates. According to adhesion measurement of the tape/photoresist interface (Fig. S6), the critical energy release rate of the photoresist/PVA substrate interface can be inferred to be lower than 43.9 J m^-2^. Overall, the energy release rate of photoresist/PVA substrate interface can be inferred within a range of 25.65 J m^-2^ to 43.9 J m^-2^ at room temperature.

In the discrete-feature configuration, the SPRR carrier forms intimate and mechanically stable contact with the SU-8 structures during pickup. Although slight local embedding of the SU-8 features into the compliant SPRR surface may occur under applied pressure, significant contact with the underlying PDMS within the feature gaps is not expected under the thickness and spacing conditions used in this work. Upon release, the SPRR elastically recovers as the load is removed, and the interfacial state approaches a near-planar configuration. For sufficiently thin SU-8 features or large feature spacing, local sagging of the carrier into the gaps could occur, potentially leading to mixed contact. While such geometric variations may influence local stress distribution, the adhesion-competition analysis in Fig. 1f, which compares the relative fracture tendencies of the SU-8/PDMS and SU-8/SPRR interfaces, remains qualitatively applicable. The planar-contact model therefore serves as a practical approximation for comparing the competing interfaces in the present configuration.

**Section S3 Influence of material components on adhesion-switching behavior of SPRR polymer**

To investigate how material components influence the adhesion-switching behavior of SPRR polymers, we prepared SPRR polymers with varying SA weight fractions (50%, 60%, and 80%) in the SA-UDA copolymer. We then fabricated multiple SPRR polymer/glass substrates. These polymers exhibit distinct changes in storage modulus between rigid and rubbery states, as shown in Fig. 1a. We observed that the SPRR polymer with a 50% SA weight fraction can’t effectively conform and pick up centimeter-scale SU-8 2010 photoresist on the PDMS-coated donor wafer. This is due to its inadequate conformality in rubbery state and insufficient locking strength in rigid state. In contrast, the SPRR polymers with higher SA weight fractions (60% and 80%) can pick up SU-8 2010 photoresist films from PDMS-coated donor substrate. This success can be attributed to their storage modulus which exhibits lower values in the rubbery state and higher values in the rigid state. Here, we focused on the adhesion behavior comparison between two SPRR polymers with 60% and 80% SA weight fractions. We conducted tape peeling tests on SPRR polymer (SA-60%) in both its rigid and rubbery states following the same method in Section S2. Figure S10a shows that the rubbery SPRR polymer with an 80% SA weight fraction exhibits greater adhesion to the photoresist. This can be attributed to a larger conformal contact area with the non-ideally flat photoresist film, when the SPRR polymer exhibits lower storage modulus in rubbery state. Figure S10b shows the results of tape peeling experiments on SU-8 2010 photoresist that was locked by the rigid SPRR polymer (SA-60%). The SPRR polymer with SA weight fractions of 60% can firmly lock the SU-8 photoresist when the tape peeling speed is below 1 mm s^-1^. However, when the tape is peeled up at 2 mm s^-1^, the photoresist is detached from the rigid SPRR polymer (SA-60%). The critical peeling speed for removing the photoresist from the rigid SPRR polymer (SA-60%) is estimated to be between 1 mm s^-1^ and 2 mm s^-1^, which is lower than that of the SPRR polymer (SA-80%), as discussed in the Section S2. This phenomenon can be explained by the following reasons:

(1) The rubbery SPRR polymer (SA-80%) has excellent softness for conformal contact with large-area continuous photoresist film due to its lower storage modulus in rubbery state.

(2) The maximized conformal contact is effectively maintained by SPRR polymer (SA-80%) utilizing its higher modulus in rigid state.

Consequently, the SPRR polymer (SA-80%) exhibits significant adhesion-switching behavior facilitating the transfer of large-area continuous photoresist films.

**Section S4 Local registration error analysis**

For transfer error analysis, the images of photoresist before and after transfer are processed (e.g. edge feature extraction) and then overlaid with assistance of the Fiji software via linear stack alignment with SIFT [35-36]. The alignment relies on the linear transform processes and has no nonlinear change on local features within images. The local registration error here is defined as the relative position difference of a photoresist structure between two registered images, including translation and rotation. The local translation of photoresist structures is defined as displacement of their geometric center, and local rotation is defined as the rotation angle of the photoresist structures around its geometric center.

Therefore, the translation vector of a transferred photoresist structure can be expressed as:

$\left(\mathbf{x},\mathbf{y}\right)=(x_{2}-x_{1},y_{2}-y_{1})=(x_{2},y_{2})-(x_{1},y_{1})$ **(S7)**

where $(x_{1},y_{1})$ and $(x_{2},y_{2})$ represent geometric center coordinates of the photoresist structure in two images. Therefore, the translation error can be calculated as:

$\left|(\mathbf{x},\mathbf{y})\right|=\sqrt{(x_{2}-x_{1})^{2}+(y_{2}-y_{1})^{2}}$ **(S8)**

Based on geometrical relationships, the equations for a specific photoresist structure can be obtained as follows:

$a_{1}\cos\theta_{1}+b_{1}\sin\theta_{1}=A_{1}$ **(S9)**

$b_{1}\cos\theta_{1}+a_{1}\sin\theta_{1}=B_{1}$ **(S10)**

$a_{2}\cos\theta_{2}+b_{2}\sin\theta_{2}=A_{2}$ **(S11)**

$b_{2}\cos\theta_{2}+a_{2}\sin\theta_{2}=B_{2}$ **(S12)**

where $a_{1}$ and $a_{2}$ are widths of a photoresist structure in two images, respectively. $b_{1}$ and $b_{2}$ respectively denote the lengths of the photoresist structure in two images. $\theta_{1}$ and $\theta_{2}$ are the angles of the photoresist structure in two images. $A_{1}$ and $A_{2}$ correspond to the widths of the minimum bounding rectangles encompassing a specific photoresist structure in two images, where the widths of the bounding rectangles are along the ***y***-axis. $B_{1}$ and $B_{2}$ respectively represent the lengths of the minimum bounding rectangle in two images, where the length of the rectangles is along the ***x***-axis.

Equations can be further derived:

$(a_{1}^{2}+b_{1}^{2})\sin\theta_{1}\cos\theta_{1}+a_{1}b_{1}=A_{1}B_{1}$ **(S13)**

$(a_{2}^{2}+b_{2}^{2})\sin\theta_{2}\cos\theta_{2}+a_{2}b_{2}=A_{2}B_{2}$ **(S14)**

Thus, the rotation angle of a photoresist structure can be expressed as follows:

$\begin{matrix} \theta(A_{1},B_{1},A_{2},B_{2},a_{1},b_{1},a_{2},b_{2}) \\ =\theta_{2}-\theta_{1} \\ =0.5\arcsin\left[\frac{2(A_{2}B_{2}-a_{2}b_{2})}{a_{2}^{2}+b_{2}^{2}}\right]-0.5\arcsin\left[\frac{2(A_{1}B_{1}-a_{1}b_{1})}{a_{1}^{2}+b_{1}^{2}}\right] \end{matrix}$ **(S15)**

$(x_{1},y_{1})$, $(x_{2},y_{2})$, $A_{1}$, $B_{1}$, $A_{2}$, $B_{2}$, $a_{1}$, $b_{1}$, $a_{2}$, $b_{2}$ can be measured in two registered images to calculate the local translation and rotation.

We also analyzed the errors in measurement. The smallest pixel size of registered images is $\varepsilon$, and thus the length measurement uncertainty is $\text{±}\varepsilon$. The error range in the local translation measurement can be estimated though following equation:

$\varepsilon_{\left|(\mathbf{x},\mathbf{y})\right|}=\pm\sqrt{\left[\sqrt{(x+\varepsilon)^{2}+y^{2}}-\sqrt{x^{2}+y^{2}}\right]^{2}+\left[\sqrt{x^{2}+(y+\varepsilon)^{2}}-\sqrt{x^{2}+y^{2}}\right]^{2}}$ **(S16)**

The error range in the rotation measurement can be estimated as follows:

$\begin{matrix} \varepsilon_{\theta}^{2}=\left[\theta(A_{1}+\varepsilon,B_{1},A_{2},B_{2},a_{1},b_{1},a_{2},b_{2})-\theta(A_{1},B_{1},A_{2},B_{2},a_{1},b_{1},a_{2},b_{2})\right]^{2} \\ +\left[\theta(A_{1},B_{1}+\varepsilon,A_{2},B_{2},a_{1},b_{1},a_{2},b_{2})-\theta(A_{1},B_{1},A_{2},B_{2},a_{1},b_{1},a_{2},b_{2})\right]^{2} \\ +\left[\theta(A_{1},B_{1},A_{2}+\varepsilon,B_{2},a_{1},b_{1},a_{2},b_{2})-\theta(A_{1},B_{1},A_{2},B_{2},a_{1},b_{1},a_{2},b_{2})\right]^{2} \\ +\left[\theta(A_{1},B_{1},A_{2},B_{2}+\varepsilon,a_{1},b_{1},a_{2},b_{2})-\theta(A_{1},B_{1},A_{2},B_{2},a_{1},b_{1},a_{2},b_{2})\right]^{2} \\ +\left[\theta(A_{1},B_{1},A_{2},B_{2},a_{1}+\varepsilon,b_{1},a_{2},b_{2})-\theta(A_{1},B_{1},A_{2},B_{2},a_{1},b_{1},a_{2},b_{2})\right]^{2} \\ +\left[\theta(A_{1},B_{1},A_{2},B_{2},a_{1},b_{1}+\varepsilon,a_{2},b_{2})-\theta(A_{1},B_{1},A_{2},B_{2},a_{1},b_{1},a_{2},b_{2})\right]^{2} \\ +\left[\theta(A_{1},B_{1},A_{2},B_{2},a_{1},b_{1},a_{2}+\varepsilon,b_{2})-\theta(A_{1},B_{1},A_{2},B_{2},a_{1},b_{1},a_{2},b_{2})\right]^{2} \\ +\left[\theta(A_{1},B_{1},A_{2},B_{2},a_{1},b_{1},a_{2},b_{2}+\varepsilon)-\theta(A_{1},B_{1},A_{2},B_{2},a_{1},b_{1},a_{2},b_{2})\right]^{2} \end{matrix}$ **(S17)**

$\begin{matrix} \varepsilon_{\theta}\approx\pm\sqrt{\left(\frac{\partial\theta}{\partial A_{1}}\right)^{2}\varepsilon^{2}+\left(\frac{\partial\theta}{\partial B_{1}}\right)^{2}\varepsilon^{2}+\left(\frac{\partial\theta}{\partial A_{2}}\right)^{2}\varepsilon^{2}+\left(\frac{\partial\theta}{\partial B_{2}}\right)^{2}\varepsilon^{2}+\left(\frac{\partial\theta}{\partial a_{1}}\right)^{2}\varepsilon^{2}+\left(\frac{\partial\theta}{\partial b_{1}}\right)^{2}\varepsilon^{2}+\left(\frac{\partial\theta}{\partial a_{2}}\right)^{2}\varepsilon^{2}+\left(\frac{\partial\theta}{\partial b_{2}}\right)^{2}\varepsilon^{2}} \\ =\pm\varepsilon\sqrt{\left(\frac{\partial\theta}{\partial A_{1}}\right)^{2}+\left(\frac{\partial\theta}{\partial B_{1}}\right)^{2}+\left(\frac{\partial\theta}{\partial A_{2}}\right)^{2}+\left(\frac{\partial\theta}{\partial B_{2}}\right)^{2}+\left(\frac{\partial\theta}{\partial a_{1}}\right)^{2}+\left(\frac{\partial\theta}{\partial b_{1}}\right)^{2}+\left(\frac{\partial\theta}{\partial a_{2}}\right)^{2}+\left(\frac{\partial\theta}{\partial b_{2}}\right)^{2}} \end{matrix}$**(S18)**

**Section S5 Adhesion** **hierarchy for successful dry lift-off**

For successful dry lift-off processes, the energy release rate at different interfaces should follow the adhesion hierarchy:

$$G_{\text{rigid SPRR polymer/film}}>G_{\text{photoresist/substrate}}$$

$$G_{\text{film/photoresist}}>G_{\text{photoresist/substrate}}$$

This hierarchy determines the fracture that occurred at the photoresist/substrate interface rather than the SPRR polymer/film and film/photoresist interfaces. The sputtered films typically have good adhesion to photoresist, demonstrating a high value of $G_{\text{film/photoresist}}$, exceeding the$G_{\text{rigid SPRR polymer/film}}$ and $G_{\text{photoresist/substrate}}$. Thus, the dry lift-off process is largely determined by the $G_{\text{rigid SPRR polymer/film}}$ and $G_{\text{photoresist/substrate}}$.

Surface treatment is an effective method for modulating interfacial adhesion. For example, coating a monolayer of 1H,1H,2H,2H-Perfluorooctyltrichlorosilane (FOTS) on a clean glass substrate through thermal evaporation can largely reduce the surface energy of glass substrates, minimizing $G_{\text{photoresist/substrate}}$ lower than $G_{\text{rigid SPRR polymer/film}}$. It would largely improve the yield of dry lift-off processes.

**S10 Supplementary Figures and Tables**


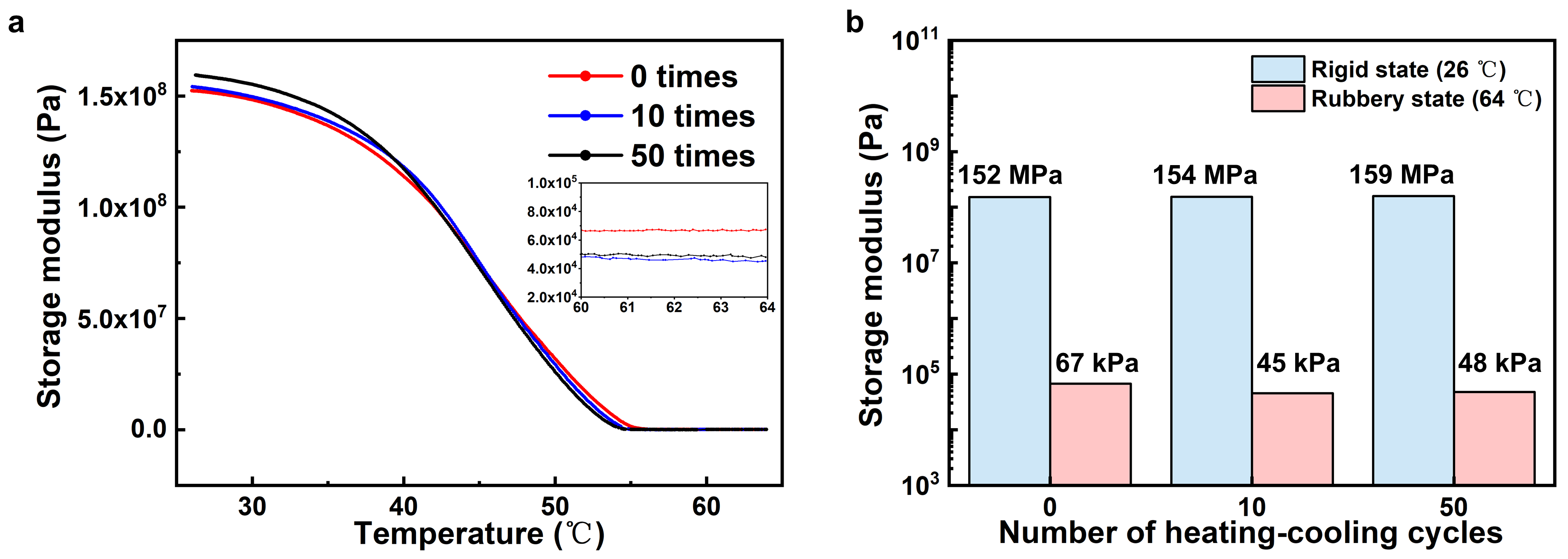


**Fig. S1** Thermomechanical characterization of SPRR polymer samples subjected to varying numbers of heating–cooling cycles. **a** Dynamic mechanical analysis (DMA) of SPRR polymer samples after different number of heating-cooling cycles. **b** Comparison of the storage modulus of SPRR polymer samples in the rigid and rubbery states


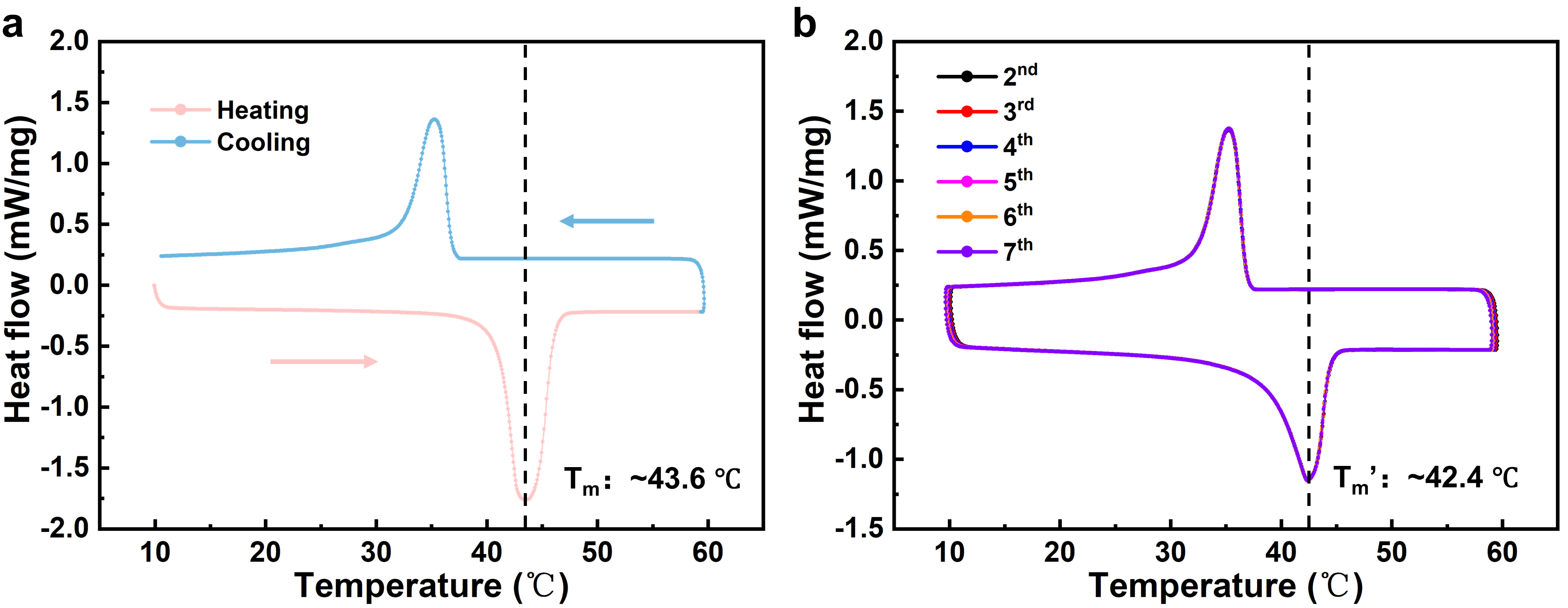


**Fig. S2** Thermodynamic characterization of the SPRR polymer by multiple differential scanning calorimetry (DSC) cycles. **a** First cycle. **b** Second to seventh cycles


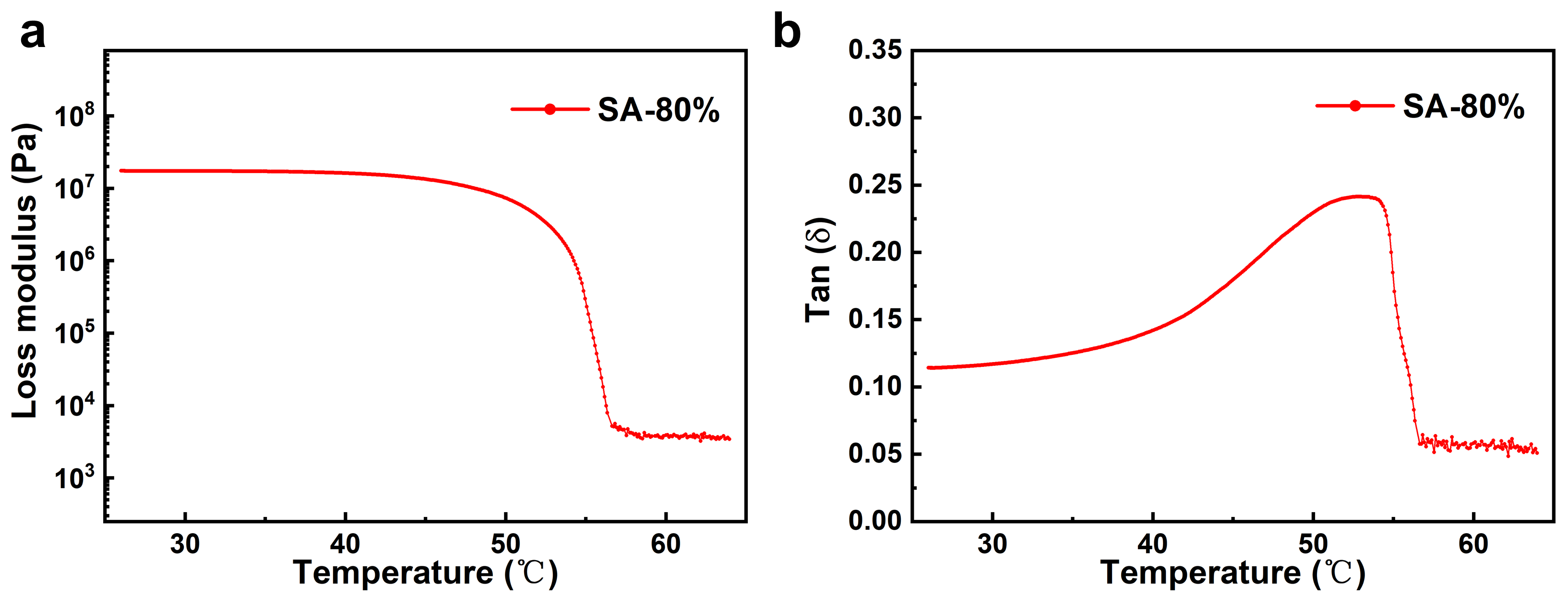


**Fig. S3** Thermomechanical characterization of SPRR polymer (SA-80%) by dynamic mechanical analysis (DMA). **a-b** Loss modulus and Tan δ of the SPRR polymer as a function of temperature


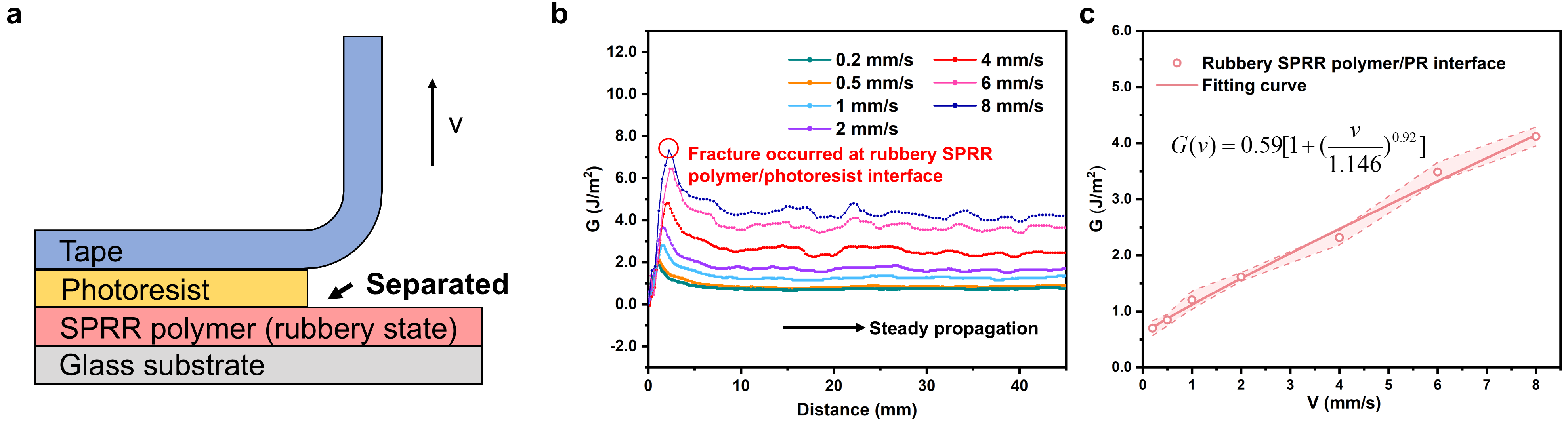


**Fig. S4** Adhesion characterization of rubbery SPRR polymer/photoresist interface. **a** Schematic of 90-degree tape peeling test on photoresist/rubbery SPRR polymer. **b** Continuous measurement of adhesion variation of rubbery SPRR polymer/photoresist interface. **c** Measured speed-dependent energy release rate of photoresist/rubbery SPRR polymer interface


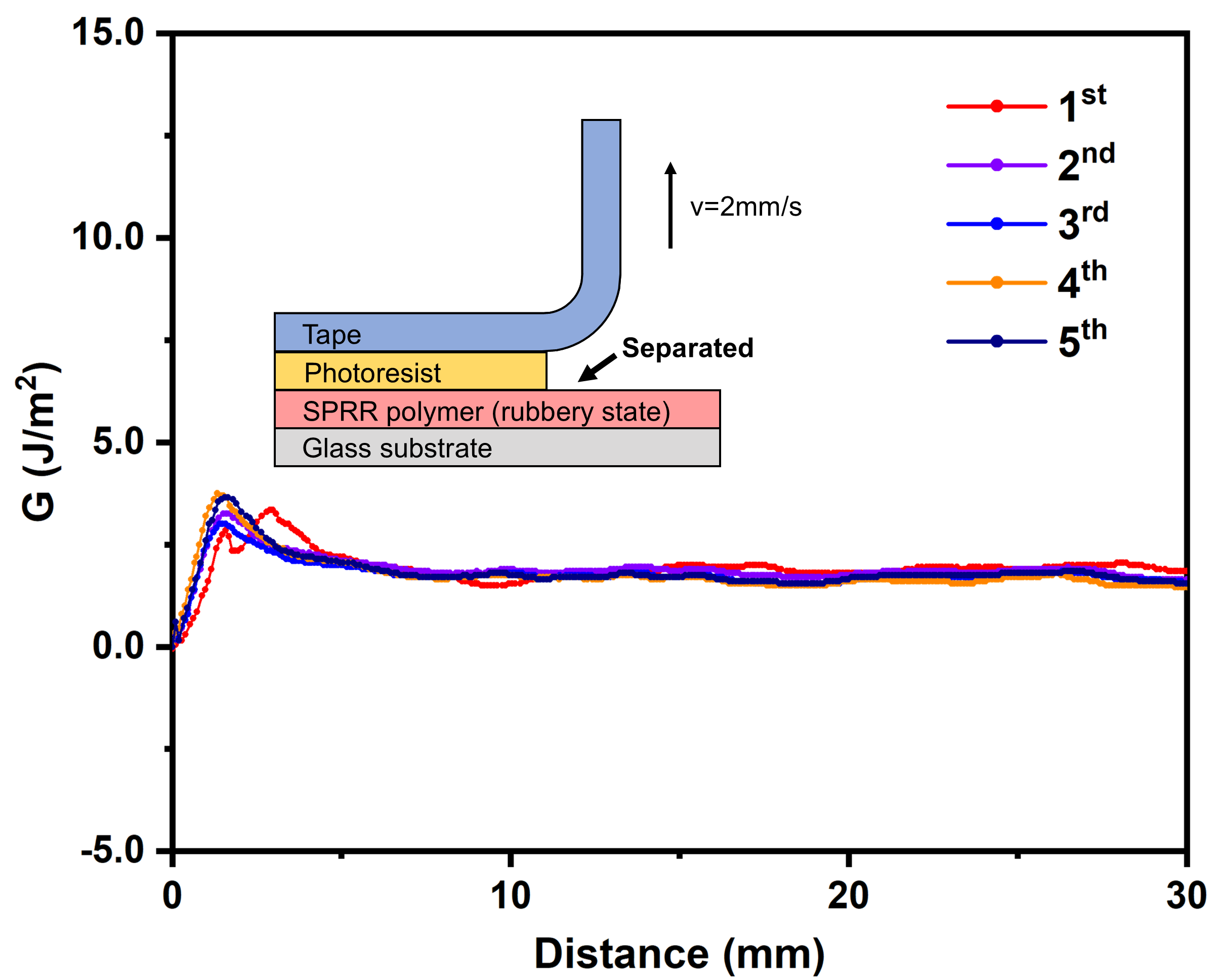


**Fig. S5** Repeated adhesion measurements of rubbery SPRR polymer/photoresist interface


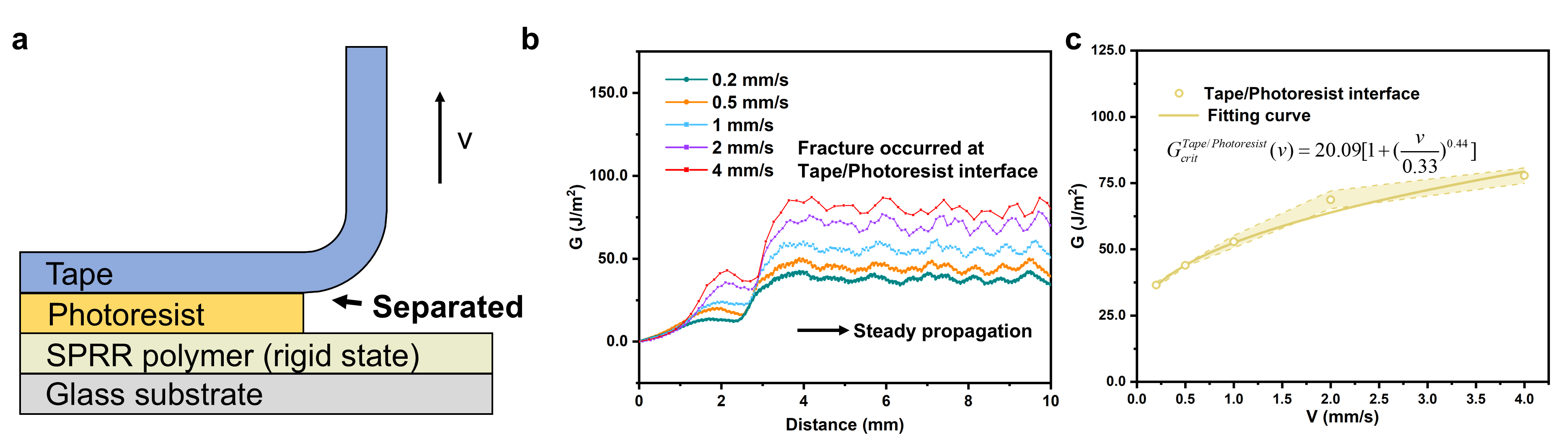


**Fig. S6** Adhesion characterization of rigid SPRR polymer/photoresist interface. **a** Schematic of 90-degree tape peeling test on photoresist/rigid SPRR polymer. **b** Continuous measurement of adhesion variation of tape/photoresist interface. **c** Energy release rate measurement of tape/photoresist interface for pickup force estimation of rigid SPRR polymer


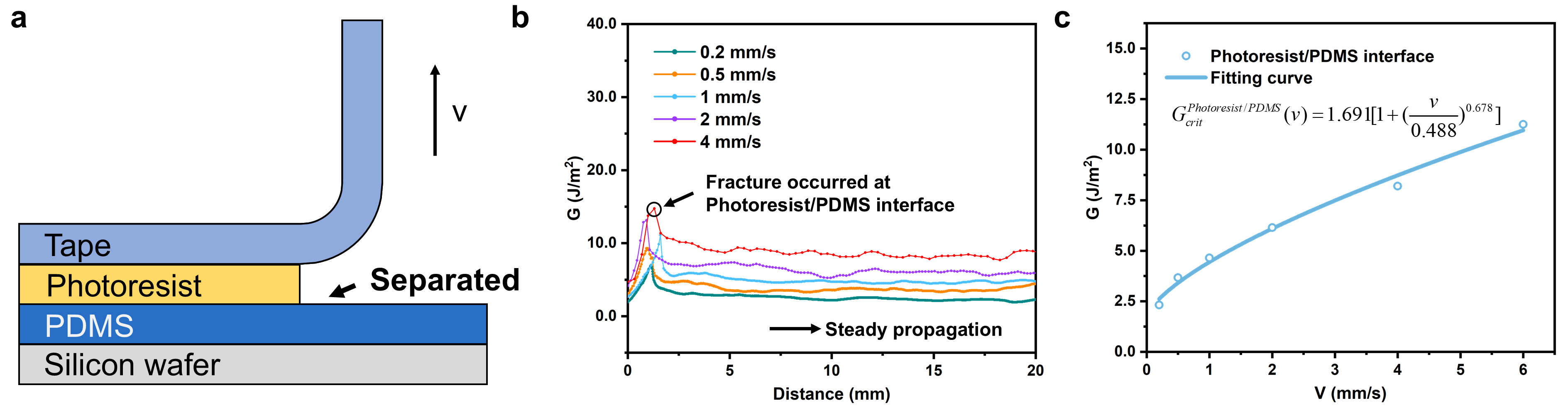


**Fig. S7** Adhesion characterization of photoresist/PDMS interface. **a** Schematic of 90-degree tape peeling test on photoresist/PDMS. **b** Continuous measurement of adhesion variation of photoresist/PDMS interface. **c** Measured speed-dependent energy release rate of photoresist/PDMS interface


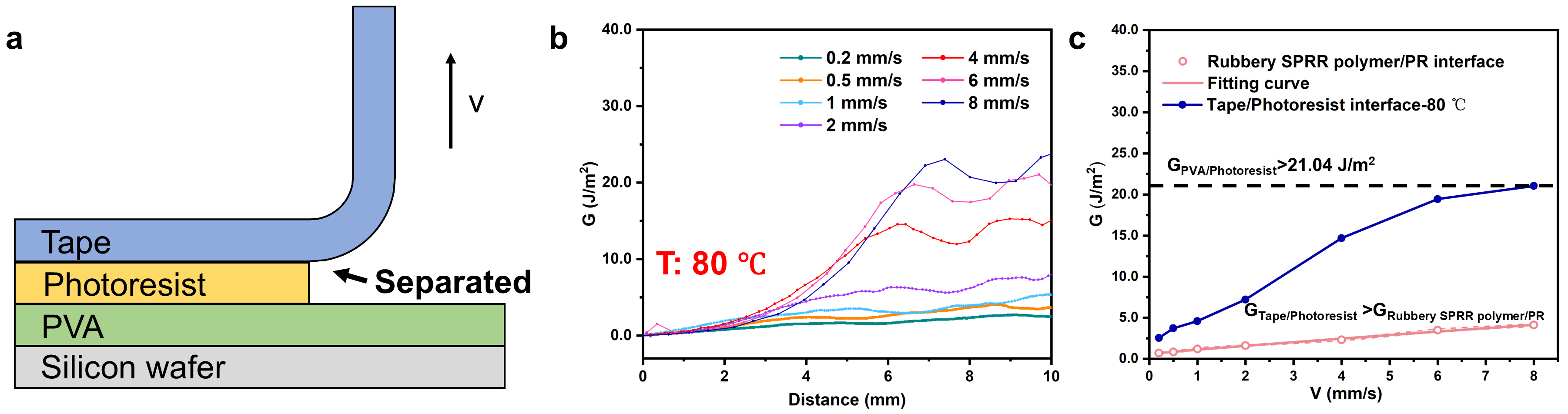


**Fig. S8** Adhesion characterization of PVA/photoresist interface at 80 ℃. **a** Schematic of 90-degree tape peeling test on photoresist/PVA substrate. **b** Continuous measurement of adhesion variation of tape/photoresist interface at 80 ℃. **c** Comparison of critical energy release rate between tape/photoresist interface and rubbery SPRR polymer/photoresist interface at 80 ℃


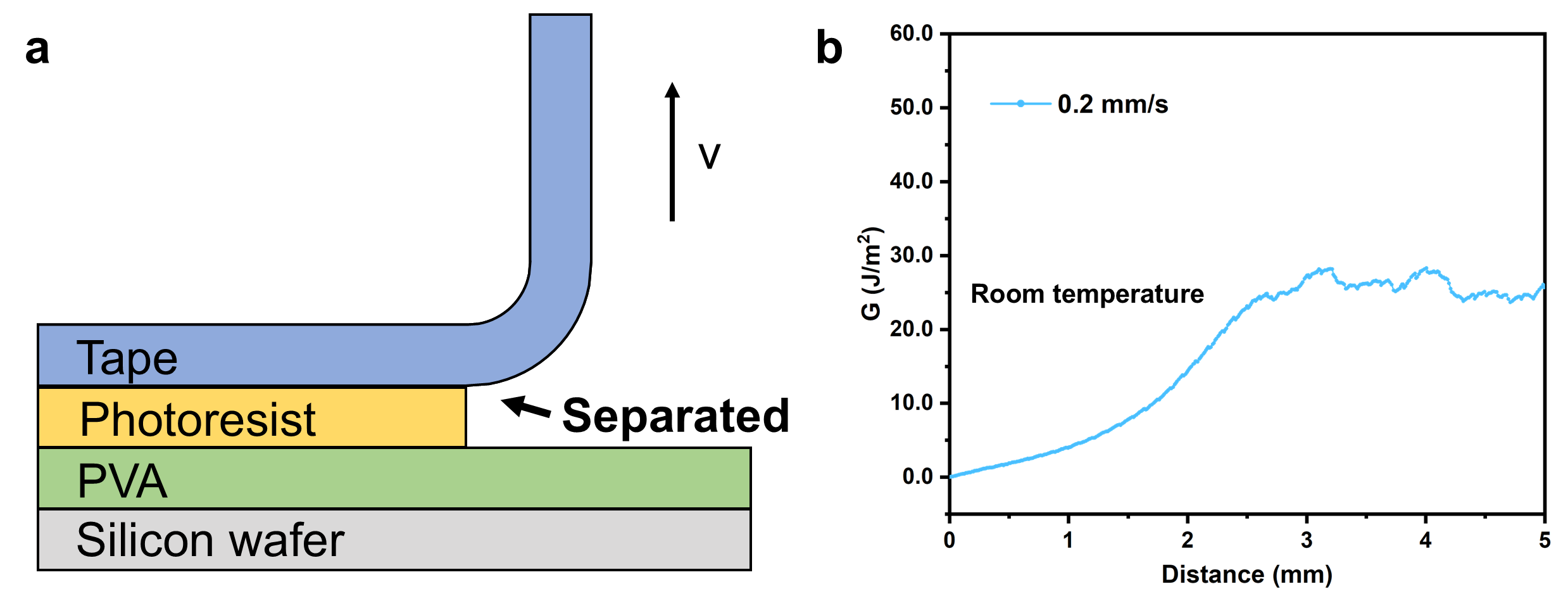


**Fig. S9** Adhesion characterization of PVA/photoresist interface at room temperature. **a** Schematic of 90-degree tape peeling test on photoresist/PVA substrate. **b** Continuous measurement of adhesion variation of tape/photoresist interface at room temperature


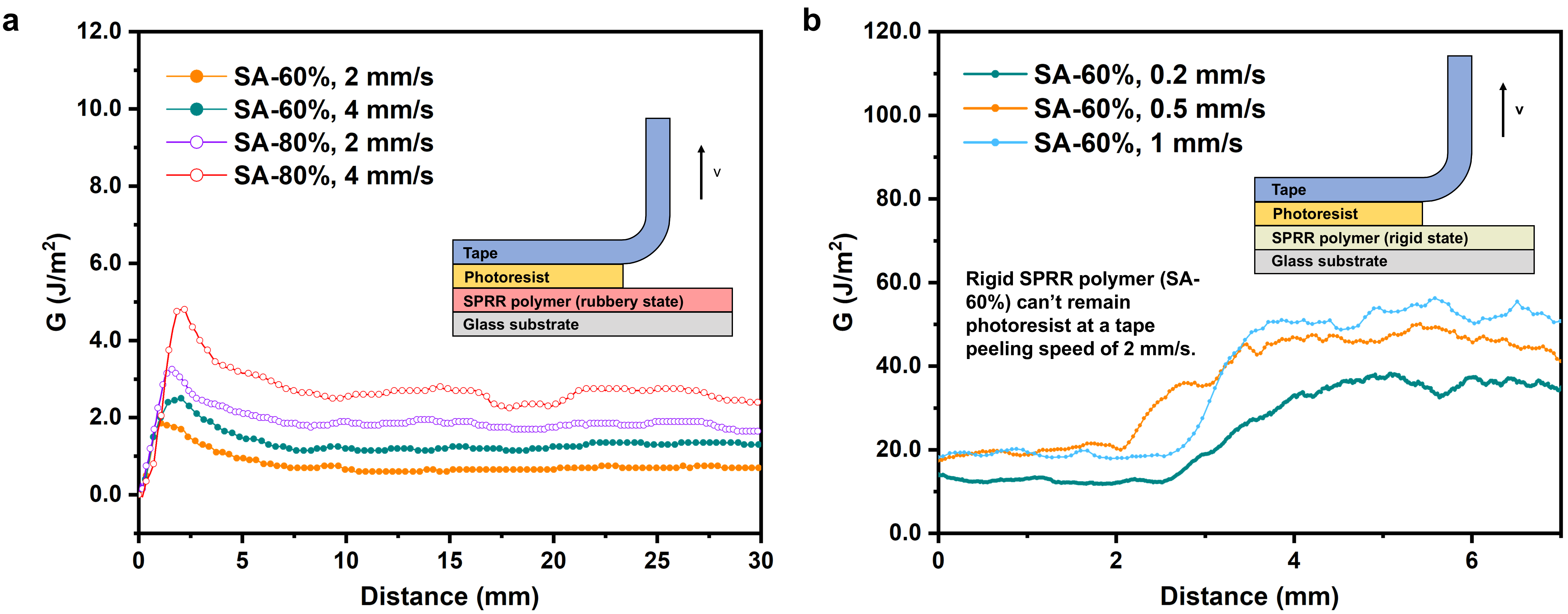


**Fig. S10** Adhesion characterization of SPRR polymer/photoresist interface with different SA weight fractions in the SA-UDA copolymers. **a** Comparison of measured adhesion variation between photoresists and rubbery SPRR polymers (weight fraction of SA in SA-UDA copolymer: 60% and 80%) during separation processes at tape peeling speeds of 2 µm and 4 µm. **b** Continuous adhesion measurement during tape peeling tests on photoresist film locked by the rigid SPRR polymer (SA weigh fraction in SA-UDA copolymer: 60%) at peeling speed of 0.2 mm s^-1^, 0.5 mm s^-1^ and 1 mm s^-1^


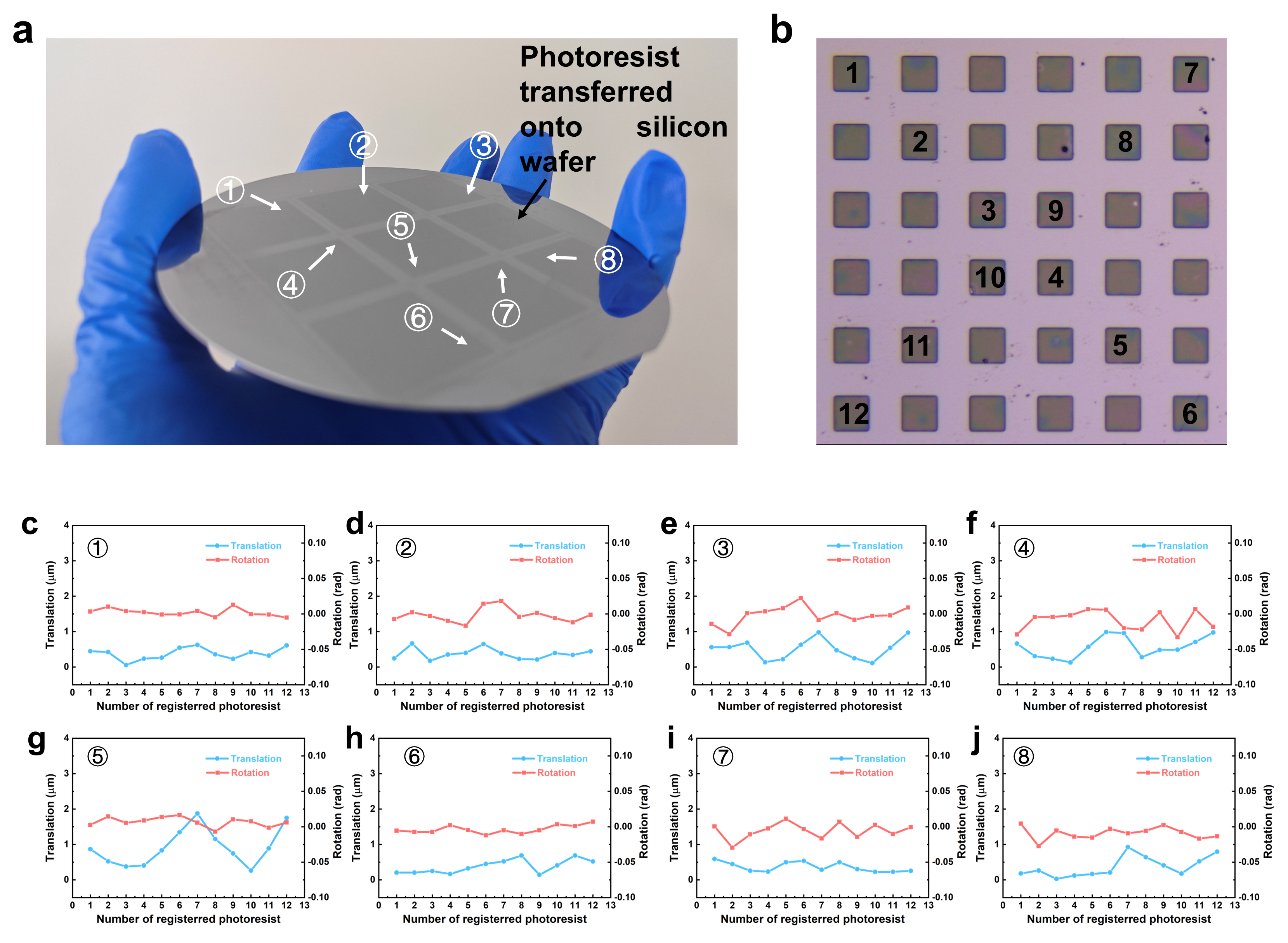


**Fig. S11** Registration analysis of photoresist before and after transfer at multiple locations on a 4-inch wafer. **a** Optical image of patterned AZ5214E photoresist array transferred onto 4-inch silicon wafer. **b** Microscope image of photoresist array after transfer. **c-j** Registration error measurement for multiple photoresist structures in several regions


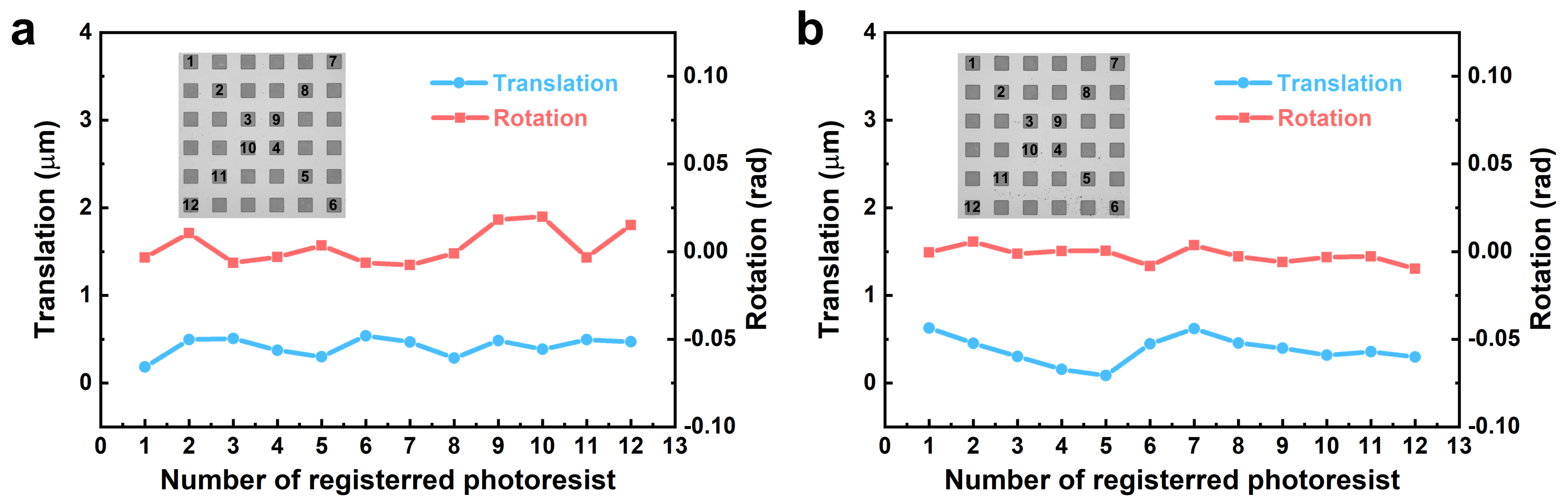


**Fig. S12** Registration error analysis of photoresist before and after transfer. The photoresists were transferred using the SPRR polymer blocks undergone **a** 10 times and **b** 50 times of heating-cooling cycles


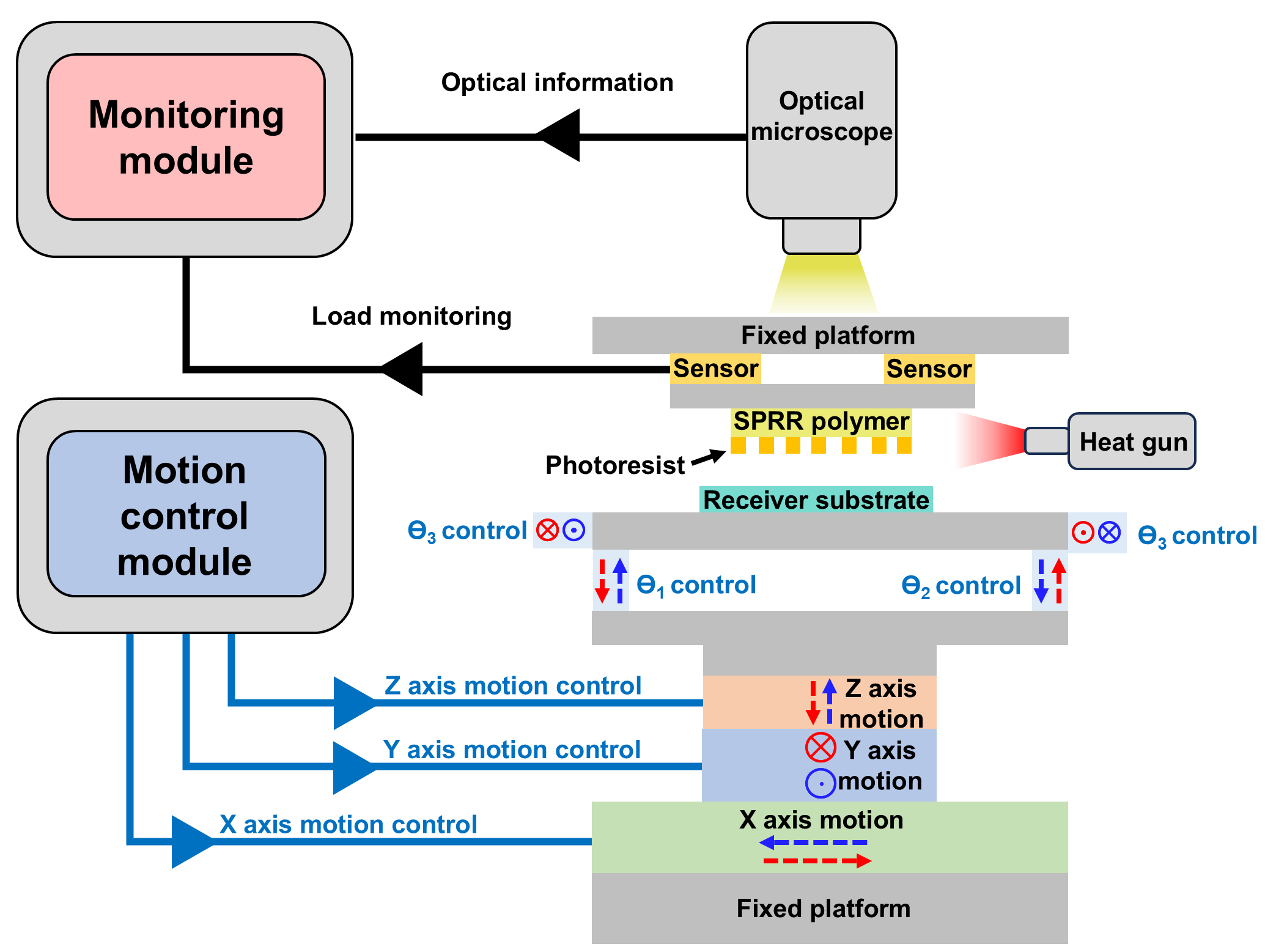


**Fig. S13** Schematic diagram of the six-axis stage


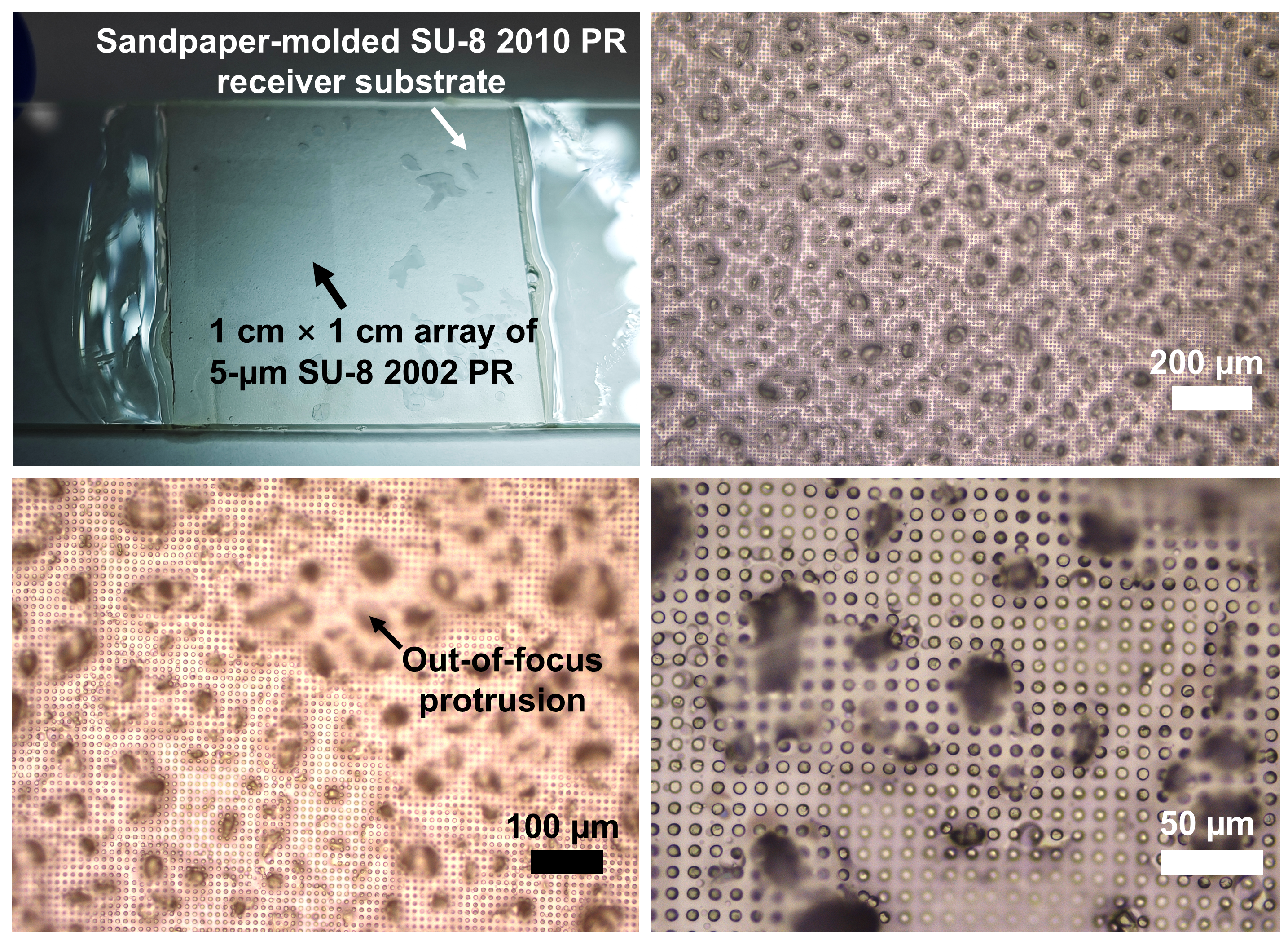


**Fig. S14** Optical images of 5-µm-diameter SU-8 2002 photoresist array transferred onto sandpaper-molded SU-8 2010 photoresist/glass substrate


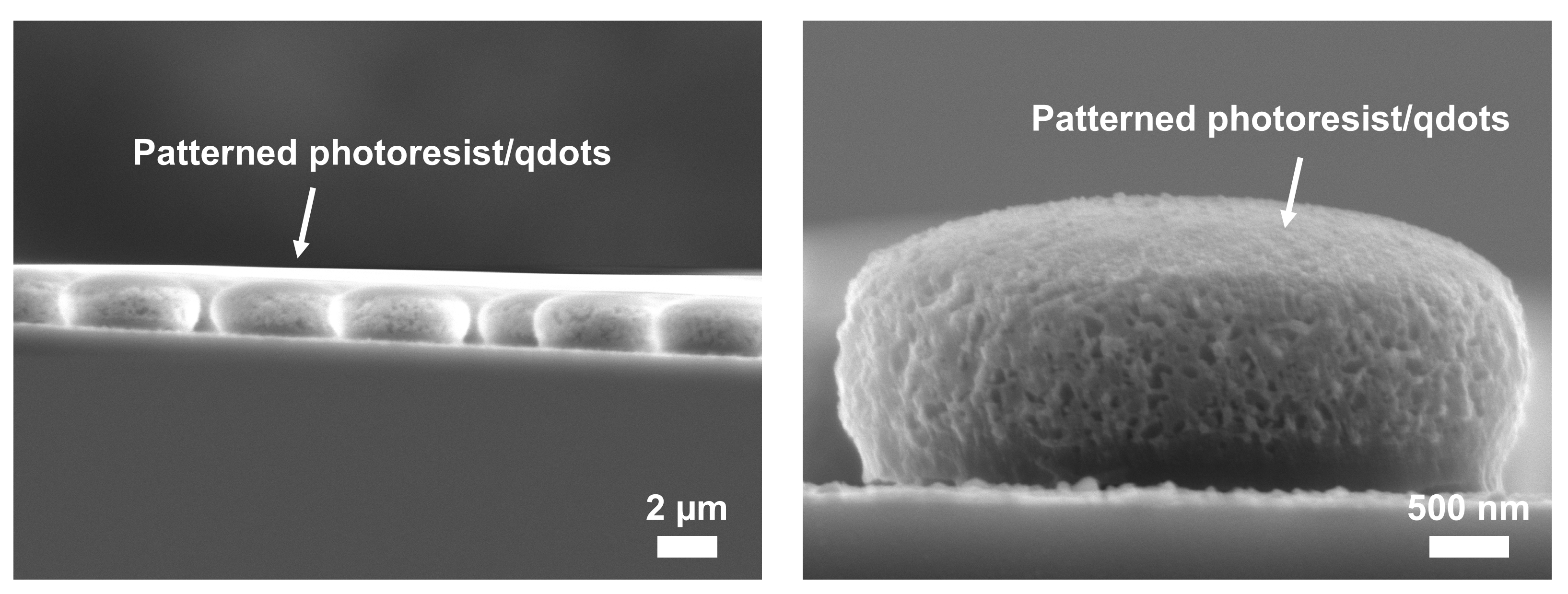


**Fig. S15** SEM images of patterned SU-8 2002 photoresist/qdots array after dry etching


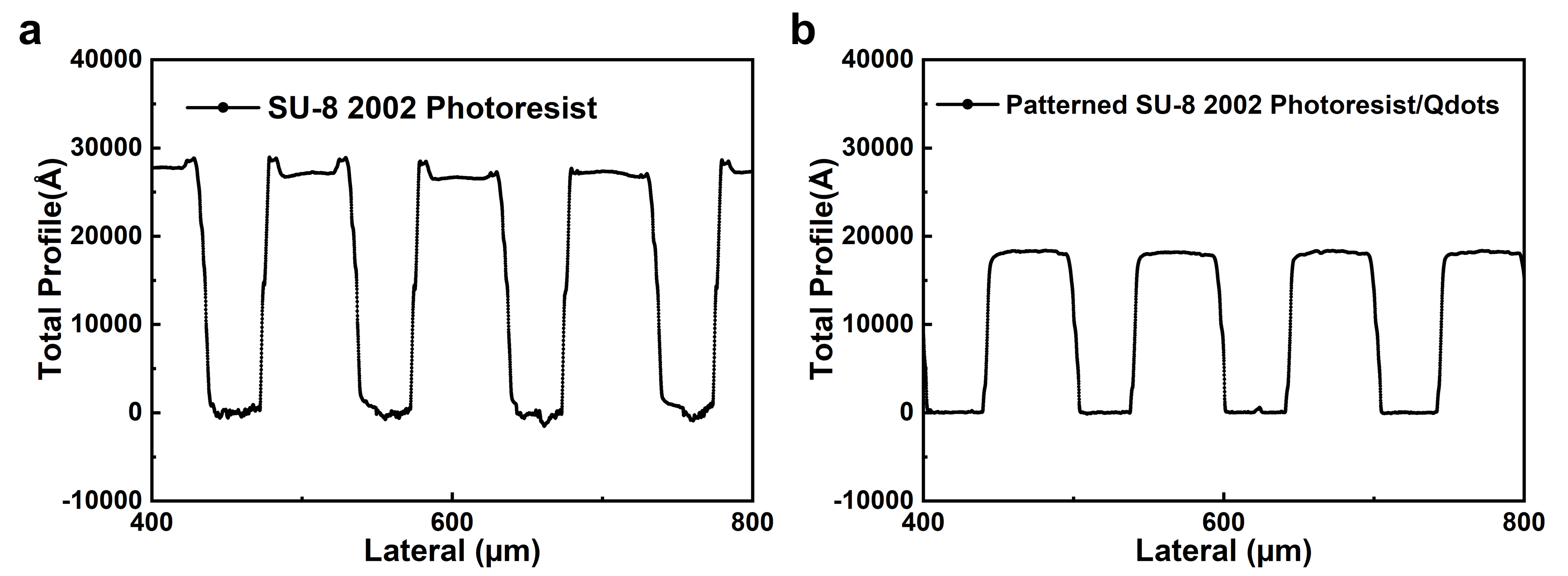


**Fig. S16** Height measurement before and after dry etching. **a** Height measurement of SU-8 2002 photoresist before dry etching. **b** Height measurement of the SU-8 2002 photoresist/qdots after dry etching


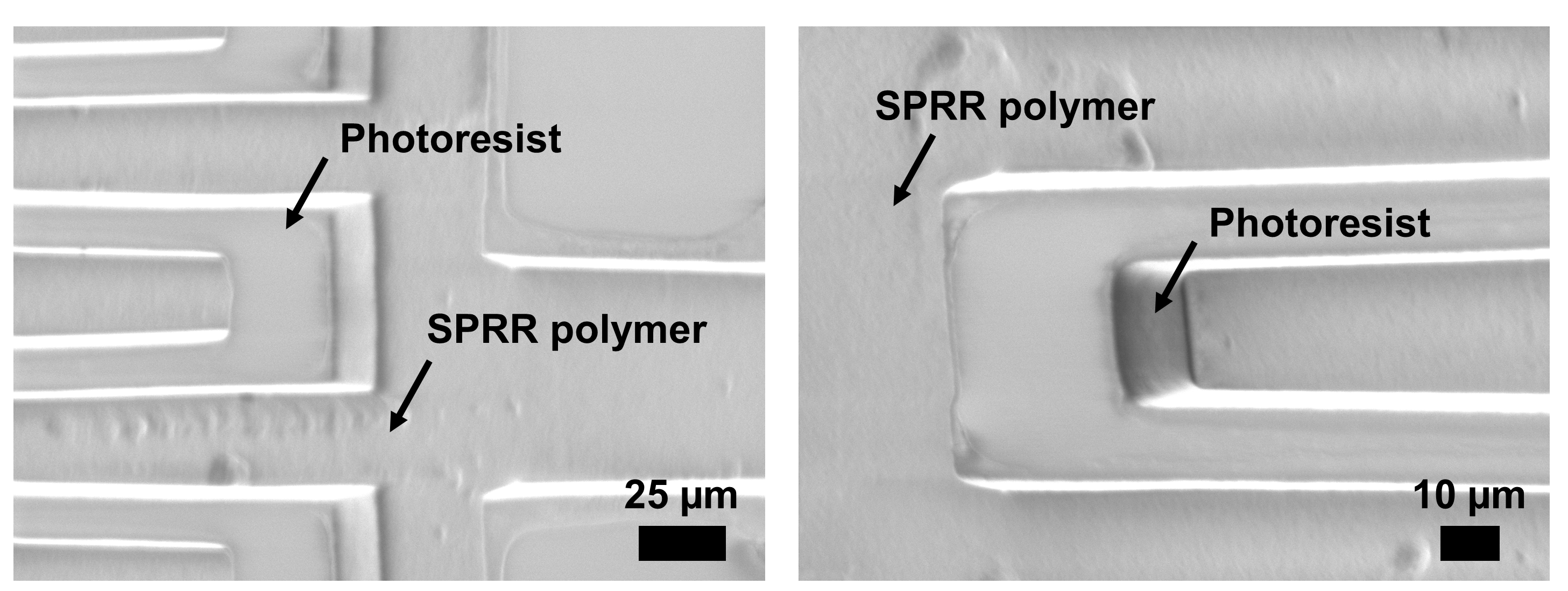


**Fig. S17** SEM images of SU-8 2010 photoresist/SPRR polymer after undergoing dry lift-off process


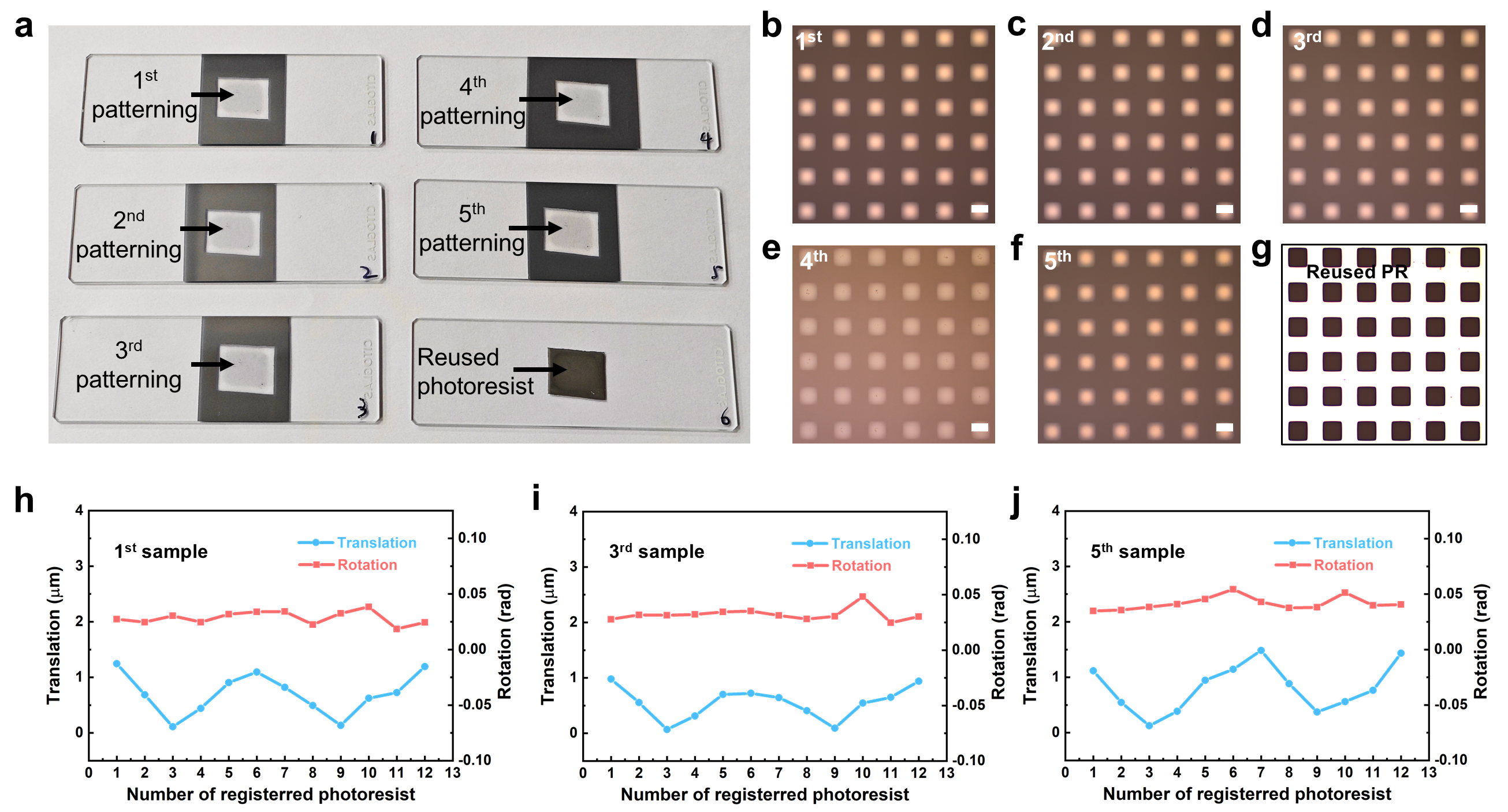


**Fig. S18** Sustainable “dry lift-off” process for metal patterning using an SPRR polymer and a reusable SU-8 2010 photoresist layer. **a** Demonstration of photoresist reuse: the same photoresist was reused for more than five cycles of dry metal patterning on glass substrates. **b-f** Optical images of a patterned Cr/Al metal array on glass substrates using the sustainable “dry lift-off” process. **g** Optical image of the photoresist after being reused for more than five cycles. **h-j** Registration analysis between the reused photoresist and the metal patterns defined in the first, third, and fifth cycles, respectively


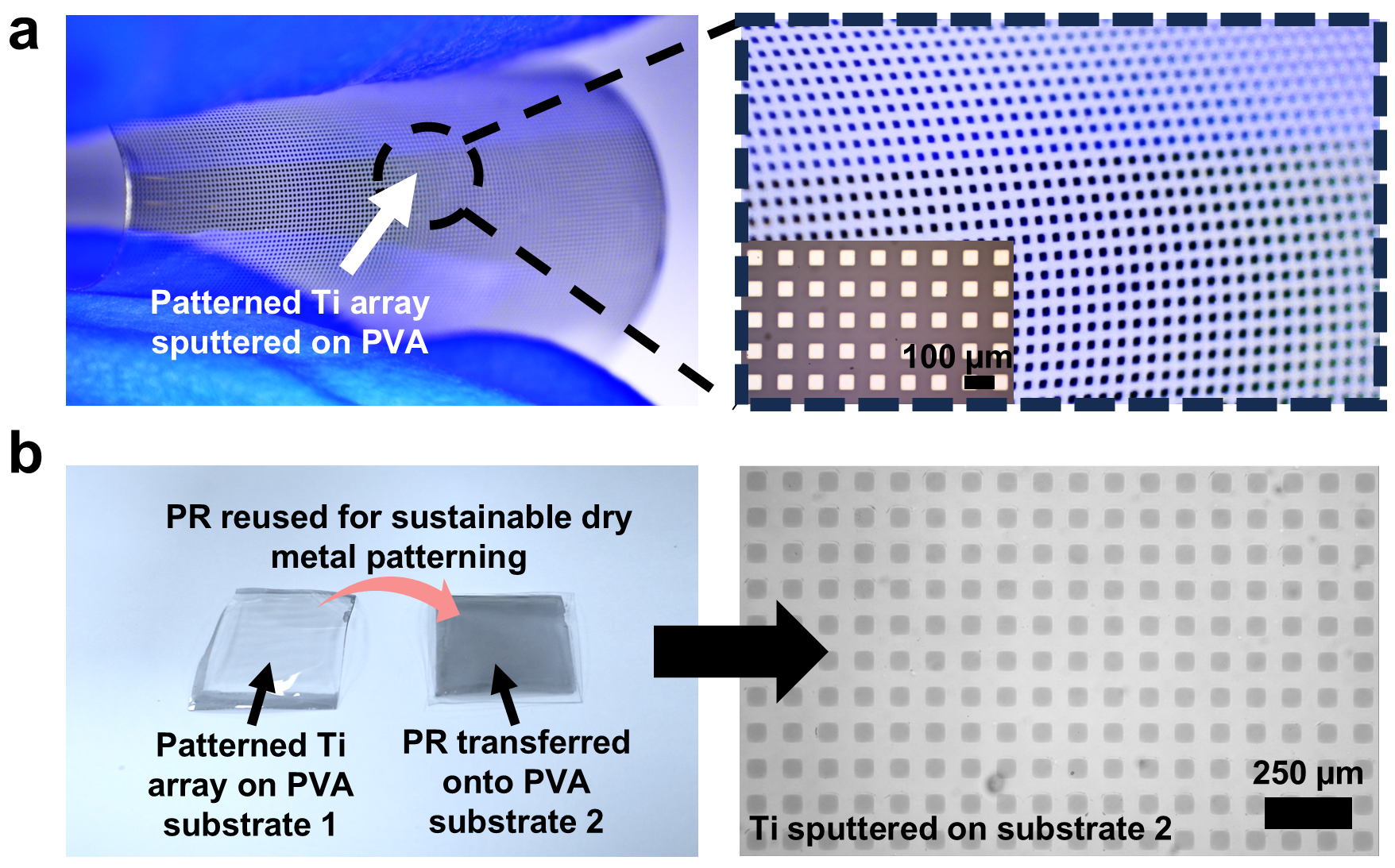


**Fig. S19** “Dry lift-off” processes for thin film patterning on water-soluble PVA substrates. **a** Optical images of a patterned array of titanium (thickness: 100 nm) on a PVA substrate using the “dry lift-off” process. **b** Demonstration of photoresist reuse: photoresist lifted from the titanium-patterned PVA substrate was re-released onto a second pristine PVA substrate, followed by the same “dry lift-off” process to define a new titanium array


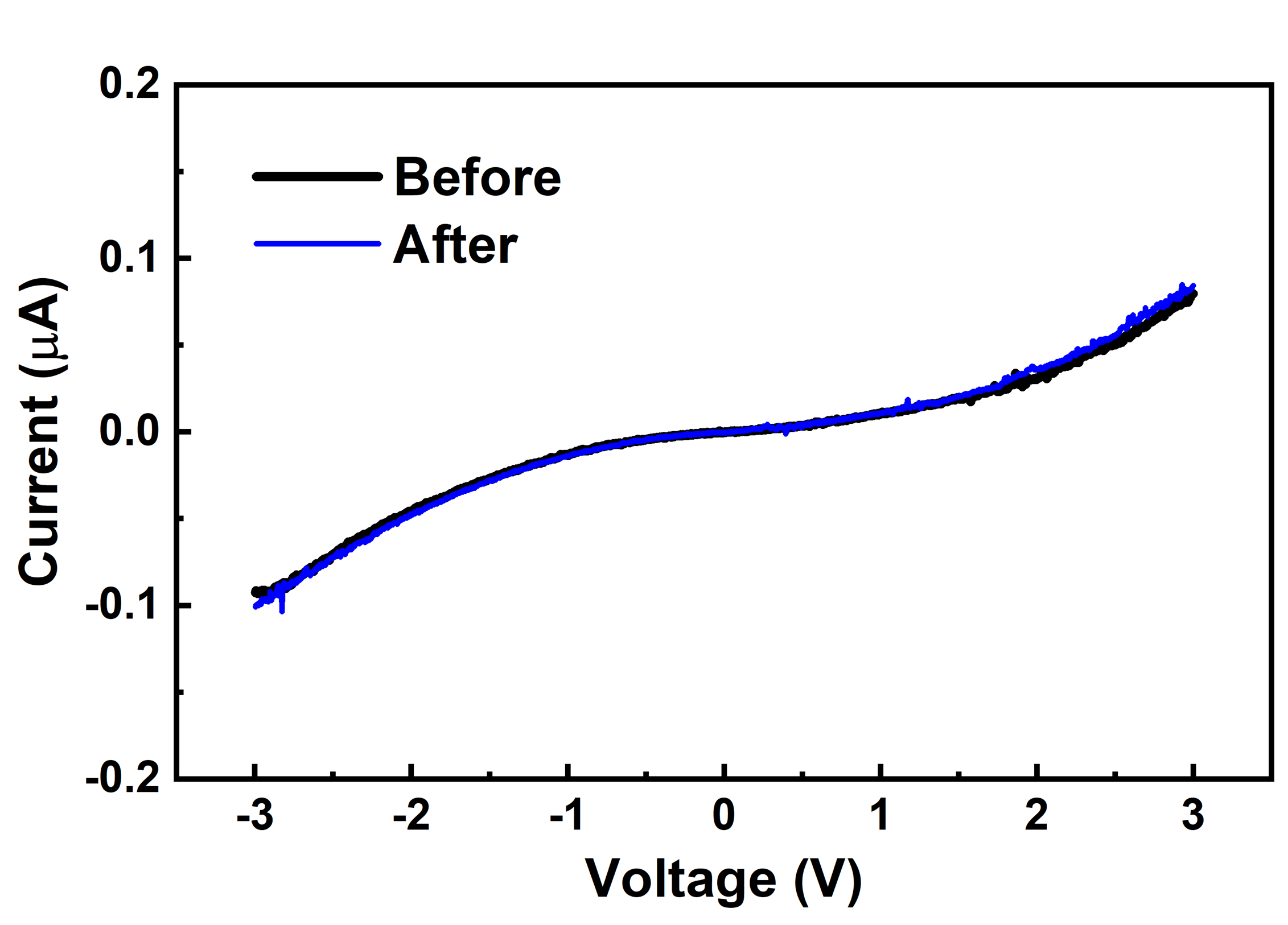


**Fig. S20** I-V response measurement of the curved photodetector before and after vibration test


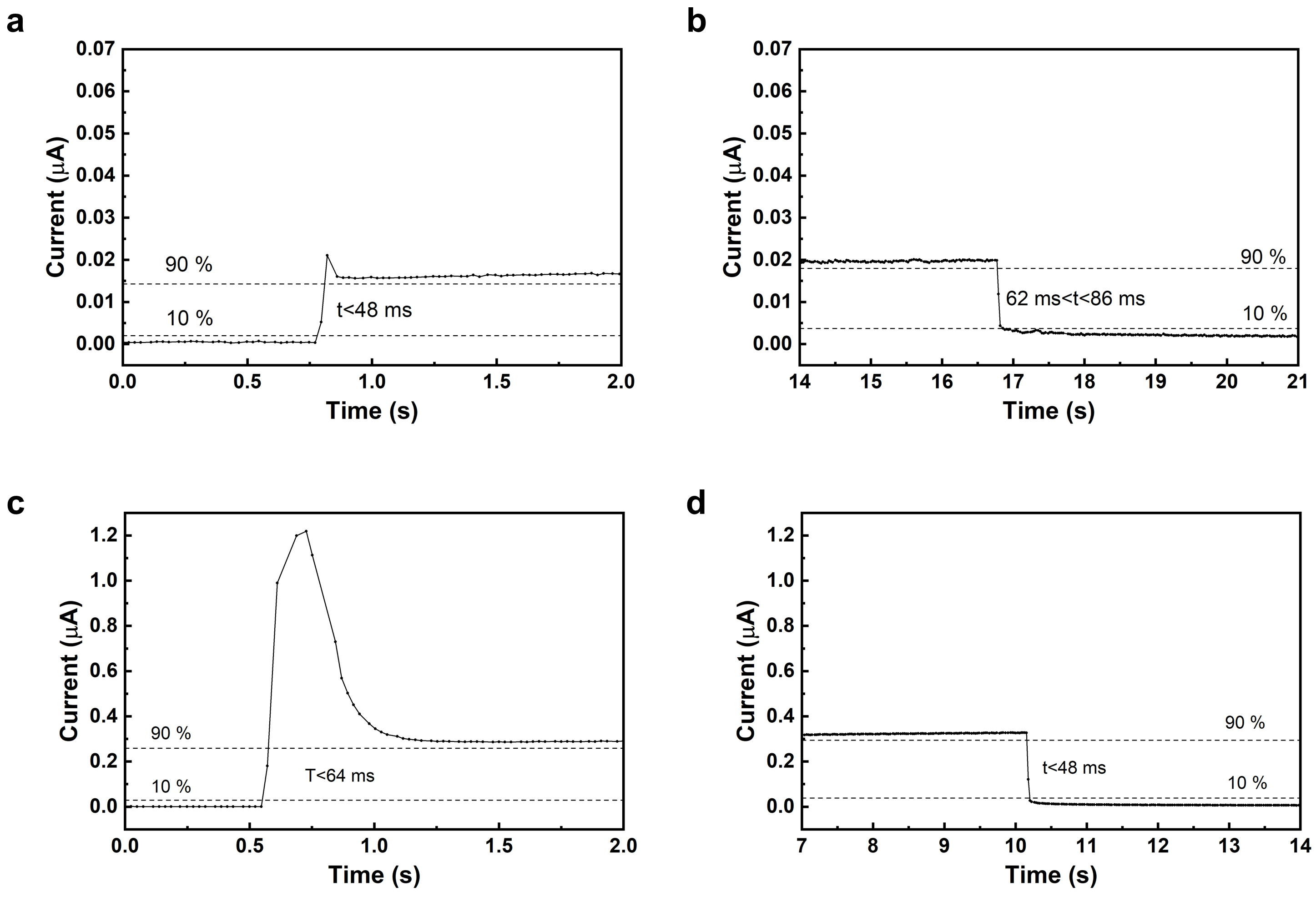


**Fig. S21** Response time characterization of the curved and planar photodetector devices with identical pattern design under on/off UV illumination (intensity: 34.1 mW/cm^2^). **a**, **b** Dynamic response curves of the curved photodetector devices fabricated using transfer lithography. **c**, **d** Dynamic response curves of the planar photodetector devices fabricated by the conventional method


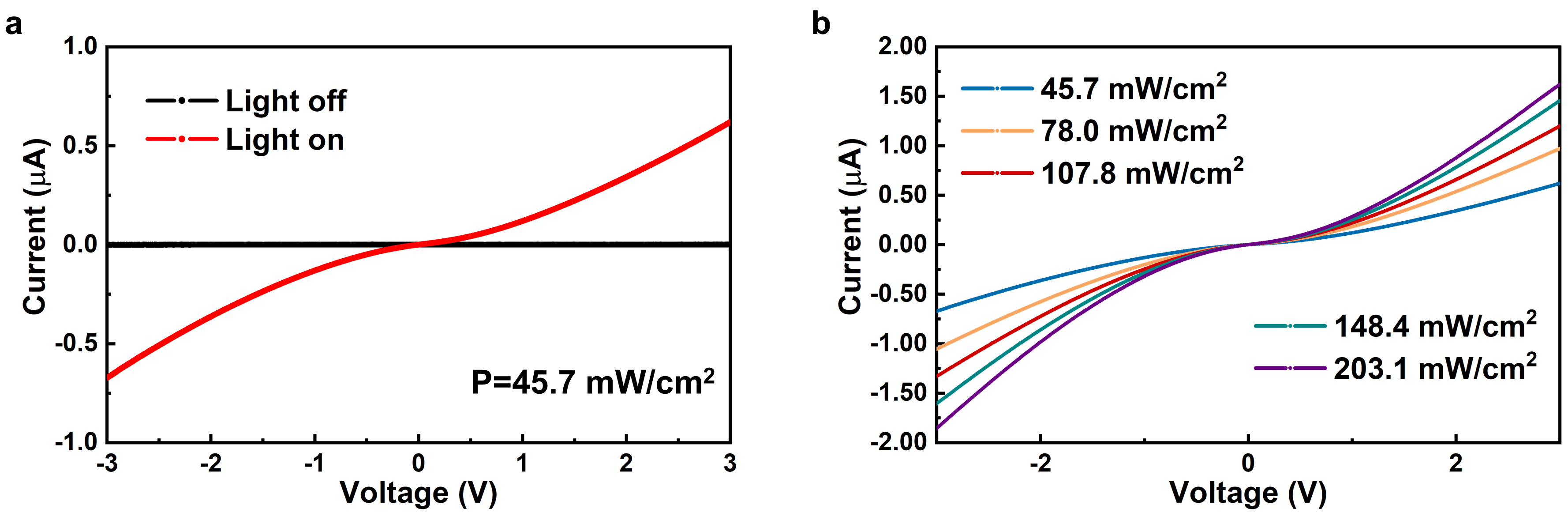


**Fig. S22** Electrical characterization of the planar photodetectors fabricated by the conventional method. **a** I–V characteristics of the planer photodetector with and without 365 nm UV illumination (power intensity: 45.7 mW/cm^2^). **b** I–V response of the planar device under varying UV illumination power intensities

**Table 1** Comparison of several methods for photoresist transfer

| Stamp | Photoresist  Compatibility | Transfer area | Thickness | Resolution | Peeling Speed | Refs |
| --- | --- | --- | --- | --- | --- | --- |
| PDMS | PMMA | 4 inches | <130 nm | 10 nm | 10 mm/s | [24] |
| Thermal release tape | Customized | 4 inches | - | 3 µm | >1 mm/s | [5] |
| SPRR polymer (SA-80%) | AZ5214E,  SU-8 2002,  SU-2010 | 4 inches | 2-10 µm | 5-50 µm | <4 mm/s  (SU-2010) | This work |
| SPRR polymer (SA-60%) | AZ5214E,  SU-8 2002,  SU-2010 | Several centimeters | 2-10 µm | 5-50 µm | <2 mm/s  (SU-2010) | This work |
| SPRR polymer (SA-50%) | AZ5214E,  SU-8 2002, | Several centimeters | 2-3 µm | 5-50 µm | - | This work |
